# Pharmacokinetic modeling and efficacy of ceftobiprole medocaril against pneumonic tularemia in cynomolgus macaques

**DOI:** 10.1128/aac.01278-25

**Published:** 2025-11-18

**Authors:** Mark M. Hahn, Michael S. Anderson, Matthew Snyder, Wantong Du, Ying-Liang Chou, Brent McCracken, Jennifer I. Smart, Karine Litherland, Stephen Keech, Franziska von Siebenthal, Mark Jones, Lisa N. Henning

**Affiliations:** 1 Battelle 42786, West Jefferson, Ohio, USA; 2 Basilea Pharmaceutica International Ltd 449688 , Allschwil, Switzerland; University of Houston, Houston, Texas, USA

**Keywords:** ceftobiprole medocaril, pharmacokinetic modeling, *Francisella tularensis*, preclinical models, pneumonic tularemia, biodefense and biothreat preparedness

## Abstract

*Francisella tularensis* is the etiologic agent of tularemia, and there are few FDA-approved antibiotics to treat infections. Ceftobiprole medocaril has potential for being developed as a therapeutic for the treatment of inhalational tularemia, as it has previously demonstrated efficacy *in vivo*. Pharmacokinetic modeling of ceftobiprole in cynomolgus macaques was used to predict an efficacious dose of ceftobiprole medocaril against inhalational tularemia in the disease model based on the measured exposures and human clinical exposure data. The efficacy of ceftobiprole medocaril against inhalational tularemia was then evaluated in the cynomolgus macaque disease model using three doses designed to mimic the human mean clinical exposure of ceftobiprole medocaril (including the predicted efficacious dose). Treatments of 0.00 (placebo), 1.33, 6.67, or 20.0 mg/kg ceftobiprole medocaril (q8h for 10 days) were administered to cynomolgus macaques by intravenous infusion after animals were exposed to aerosolized *F. tularensis* and exhibited a fever. Control animals exhibited a 12.5% survival rate, a median time to death of 11.63 days post-challenge, and significantly elevated tissue burdens. By comparison, animals receiving 1.33 mg/kg treatments with ceftobiprole medocaril exhibited a 50% survival rate, and animals receiving either of the higher doses exhibited an 87.5% survival rate. Evidence of adaptive immunity (seroconversion) was identified in all surviving animals, though detection of *F. tularensis* with sensitivity to ceftobiprole *in vitro* in a limited number of treated and surviving animals indicates that the clinical efficacy of ceftobiprole medocaril may be enhanced by treatment durations greater than 10 days.

## INTRODUCTION

*Francisella tularensis* is a highly infectious pathogen that causes tularemia in humans and animals. *F. tularensis* subspecies *tularensis* Schu S4 can cause lethal cases from inhalation of fewer than 10 CFUs (1). The high infectivity rate, environmental stability, and ability of *F. tularensis* to be cultured in large quantities have caused *F. tularensis* to be categorized by the Centers for Disease Control and Prevention as a priority category-A threat agent with a significant risk for use as a biological weapon (2). Therefore, it is necessary to have redundant antibiotic indications for the treatment of *F. tularensis* in disease manifestations that are likely to be encountered following intentional release, such as inhalational tularemia (3). Ideally, the clinical resources available would represent multiple mechanisms of action to mitigate the risk of natural or engineered resistance to various antibiotic classes (4, 5). However, doxycycline is the only antibiotic currently approved by the US Food and Drug Administration (FDA) for treating tularemia. One potential alternative therapy for the treatment of tularemia is ceftobiprole medocaril (the prodrug of ceftobiprole).

Ceftobiprole medocaril has an established clinical safety and efficacy profile (6–10), including treatment for certain bacteremias (11), right-sided infective endocarditis (11), acute bacterial skin and skin-structure infections (11), hospital-acquired bacterial pneumonia (excluding ventilator-associated pneumonia) (12, 13), and community-acquired bacterial pneumonia (14, 15), with several of these indications gaining recent approval in the USA by the FDA (11). Furthermore, ceftobiprole medocaril was previously tested against inhalational tularemia in the Fisher 344 rat model of disease (16), and was demonstrated to be an effective treatment when administered by infusion at a dosing regimen predicted to mimic the human clinical dosing-equivalent exposure (145 mg/kg ceftobiprole medocaril, q8h [infused over 1 h] for 14 days beginning 3 days post-challenge) (17). Rats treated with ceftobiprole medocaril exhibited a 92% survival rate (equivalent to levofloxacin-treated rats and significantly different from placebo-treated rats [0% survival]), and no evidence of *F. tularensis* infection was identified in lung, blood, liver, or spleen samples collected from animals surviving 31 days post-challenge.

With the efficacy of ceftobiprole medocaril against inhalational tularemia demonstrated in rats, further evaluation in a large animal disease model represents a critical step in the development of ceftobiprole medocaril for potential approval as an indication against pneumonic tularemia by the FDA Animal Rule (18). The cynomolgus macaque (CM) model of pneumonic tularemia is qualified by the FDA (19), strongly represents the characteristics and course of the human disease, and thus represents the optimal large animal testing system for ceftobiprole medocaril. As ceftobiprole medocaril had not previously been evaluated in CMs, a pharmacokinetic (PK) profile of both the prodrug and active pharmaceutical ingredient (API) was needed to establish doses that would represent the upper and lower bounds of the human mean clinical exposures prior to evaluating the drug in the disease model. The PK profile was characterized by measuring ceftobiprole medocaril and ceftobiprole plasma concentrations in female and male CMs following an ascending dose regimen (1.33, 6.67, or 33.3 mg/kg ceftobiprole medocaril) administered by single intravenous (IV) infusions. Analysis identified the following three dosing regimens for testing against inhalational tularemia: 1.33, 6.67, or 20.0 mg/kg ceftobiprole medocaril by 2 h IV infusions, q8h for 10 days.

By establishing a PK profile of ceftobiprole medocaril and ceftobiprole in CMs and performing comparative modeling to the human clinical exposures, we identified three dosing regimens that were hypothesized to demonstrate a dose-response relationship in the efficacy of post-exposure treatment of inhalational tularemia. To test our hypothesis, we challenged CMs with aerosolized *F. tularensis* Schu S4 and evaluated the outcomes of CMs when treated with ceftobiprole medocaril at each of the three dosing regimens or with a placebo (vehicle control) after confirmation of fever (individualized to each CM). Herein, we demonstrate for the first time that ceftobiprole medocaril reduces mortality, limits severe clinical disease, and (in conjunction with innate and adaptive immunity) supports clearance of *F. tularensis* infection in the CM model. By evaluating multiple dosing regimens, we have also established minimum dosing requirements and provided clear direction on ways to further enhance the efficacy of ceftobiprole medocaril against *F. tularensis* infections.

## MATERIALS AND METHODS

### Animals

This study used a total of 38 CMs (*Macaca fascicularis*) (21 male and 17 female) (Inotive, Indianapolis, IN). Body weights for each animal at initial use (dosing or challenges) ranged from 2.5 to 5.0 kg. This study was conducted in two phases. Phase I (PK modeling) used six non-human primates (NHPs) (three male and three female), and phase II (treatment evaluation) used 32 NHPs (18 male and 14 female). General procedures for animal care and housing met AAALAC International recommendations as described in The Guide for the Care and Use of Laboratory Animals (20) at the time of conduct. Water was available *ad libitum,* and food was provided daily based on individual animal weights.

Each NHP was surgically implanted with two vascular access ports (VAPs; AVA Biomedical, Wilmette, IL), and phase II animals were also implanted with a telemetry transmitter (model M00-F1; Data Sciences International, St. Paul, Minnesota) by the animal vendor. Health screenings were conducted to confirm negative results for prior exposure to *Mycobacterium tuberculosis*, simian immunodeficiency virus, simian T-cell leukemia virus 1, and herpes B virus (*Macacine alphaherpesvirus* 1). Additionally, each animal was tested for simian retrovirus (SRV1 and SRV2) and *Trypanosoma cruzi* by PCR and ELISA. Animals for phase II were also screened for *Salmonella*, *Shigella*, and intestinal parasites by fecal culture and prior *F. tularensis* exposure by serology. Animals were quarantined and acclimated to the BSL-2 (phase I) and BSL-3 (phase II) facilities and equipment prior to research activities. Animals enrolled in phase II were not treated with any antibiotics or anti-parasitics within 14 days of exposure to *F. tularensis* and were confirmed to have a white blood cell (WBC) count <20.0 × 10^3^ cells/µL and a C-reactive protein (CRP) concentration of <2.5 mg/dL within 14 days of exposure in line with model guidelines (19).

### Pharmacokinetic analysis (phase I)

#### Ceftobiprole medocaril formulation

All antibiotic and formulation reagents were pharmaceutical grade. Ceftobiprole medocaril was prepared once for each round of testing in 5% dextrose (wt/vol) solution for injection (ICU Medical, Lake Forest, IL). Briefly, ceftobiprole medocaril, 500 mg vials (Nipro Pharma Corporation; Osaka, Japan; lot number 20P03) (containing 706 mg of ceftobiprole medocaril sodium) were stored at 2°C–8°C until reconstitution and dilution. To reconstitute, 10 mL of vehicle was injected into each stock vial using a sterile needle and syringe to pierce the rubber stopper. Once dissolved by shaking (up to 10 minutes), vials were placed at rest to allow foam to dissipate, visually inspected to ensure the absence of particulate matter, then stock solutions were further diluted in vehicle to the target concentration (described below) and mixed by gently inverting. Dilute doses were stored in infusion cassettes at 2°C–8°C, protected from light, and were used for infusions within 36 h of dilution (including infusion time).

#### Antibiotic administration

There were three rounds of testing in phase I, with each round delivering a single IV infusion lasting 2 h to each animal. Each round was separated by a washout period of ≥6 days and delivered a dose of ceftobiprole medocaril greater than that of the previous round. The following doses of ceftobiprole medocaril were administered: 1.33 mg/kg (infused as a 0.2 mg/mL solution), 6.67 mg/kg (infused as a 1.0 mg/mL solution), and 33.3 mg/kg (infused as a 5.0 mg/mL solution), based on individual animal body weights measured the day before dosing. Based on predicted dissociation rates of the API, the nominal ceftobiprole doses were considered to be 1 mg/kg, 5 mg/kg, and 25 mg/kg, respectively. Infusions were delivered via a CADD Legacy Plus ambulatory infusion pump connected to the jugular VAP with a Cath-in-Cath infusion line.

#### Blood collection and plasma processing

Whole blood was collected from femoral VAPs prior to infusion starts and at the following time points relative to infusion stop times: 5 minutes; 0.5, 1, 2, 4, 6, 8, and 12 h. Whole blood was immediately added to K_3_-EDTA tubes and acidified with citric acid (19.8 µM final concentration). Plasma was separated from samples by centrifugation at 2°C–8°C and frozen at -85°C to −60°C until analyzed.

#### Bioanalytical evaluation

Each plasma sample was analyzed by LC-MS/MS to determine the concentration of BAL5788 (ceftobiprole medocaril prodrug) and BAL9141 (ceftobiprole active moiety). Working on wet ice, internal standard working solution (80 µL of BAL5788-d_4_ and BAL9141-d_4_ in 80:20 methanol:0.5% heptafluorobutyric acid) was added to all plasma samples, matrix blank (blanks with internal standard) samples, matrix calibration standards, and matrix quality control (QC) samples, except for all matrix double blank (blanks without internal standard) samples. A total of 80:20 methanol:0.5% heptafluorobutyric acid (80 µL) was added to all matrix double blank samples. After vortexing at high speed for 1 minute, the samples were centrifuged for 10 minutes at 8°C and 14,000 rpm. The resulting supernatants were transferred (80 µL) into wells of a 1 mL 96-well plate and then analyzed by high-performance liquid chromatography coupled to a tandem mass spectrometric detector (chromatographic system settings are described in Table S1). System suitability was determined by injecting a lower limit of quantification (LLOQ) standard followed by a matrix double blank sample before each data set. Sensitivity, retention time, and carryover were evaluated. The standard showed symmetrical peak shapes and expected retention times. The signal-to-noise ratio for BAL5788 and BAL9141 was ≥5:1. The response of the analytes in the matrix double blank was ≤20% of the response in the LLOQ standard, and the response of the internal standards was ≤5%.

#### Pharmacokinetic analysis

PK parameters for plasma were calculated using non-compartmental analysis in Phoenix WinNonLin 6.4 using nominal time points.

### Therapeutic efficacy analysis (phase II)

Phase II evaluated the efficacy of ceftobiprole medocaril in CMs following inhalational exposure to *F. tularensis* Schu S4. Animals were subdivided into three treatment groups of *n* = 8 CMs and one group of *n* = 8 control/vehicle-treated CMs (approximately equal numbers of males and females per group). Treatment doses were based on phase I PK analysis and modeling. The day of inhalational exposure was considered day 0 (phase II animals were challenged on 1 of 2 exposure days, assigned randomly, since aerosol challenge could not be accomplished in a single day).

#### Pre-exposure temperature monitoring

To establish baseline values and circadian rhythms for individual NHPs, telemetry data were collected for 30 seconds every 15 minutes from 10 days prior to exposure to 2 days prior to exposure. Thirty-second interval data were smoothed to four data points per hour, then averaged to define each animal’s baseline body temperature relative to 15 minute time intervals in a 24 h cycle.

#### Post-exposure temperature monitoring

Telemetry data were collected for 30 seconds every 15 minutes after exposure, and collection continued through study termination. For each 30 second sampling period, data were smoothed to a single data point for comparison to the baseline temperature at the corresponding interval. Body temperature was utilized to determine when treatment was initiated for each CM, with the specific trigger of fever (defined as ≥2 consecutive hours of a body temperature ≥1.5°C in excess of baseline [inclusive], consistent with previous uses of the model [21, 22]).

Animals that experienced hypothermia (defined as ≥2 consecutive hours of a body temperature ≥1.5°C less than the lowest of all baseline values [inclusive]) were anesthetized and euthanized immediately.

#### Preparation of *F. tularensis* for aerosol challenge

A submaster cell bank of *F. tularensis* subspecies tularensis strain Schu S4 (BEI Resources, Manassas, VA; catalog number NR-10492) was used for infectious challenge material. Bacteria were prepared and analyzed for challenge as previously described (21).

#### Inhalational challenge

NHPs were anesthetized and exposed to aerosolized *F. tularensis* using a large animal exposure system with a head-only exposure chamber as previously described (23), except that the aerosol particle size distribution was determined using an Optical Particle Sizer (Model 3330, TSI Inc., Shoreview, MN) with a diluter (Model 3332-100, TSI Inc., Shoreview, MN). Animals were challenged at a target inhaled dose of 1,000 CFUs/CM of *F. tularensis* Schu S4, and body plethysmography was performed in real-time to measure tidal volume, total accumulated tidal volume, and minute volume. Actual exposure doses delivered to each NHP were calculated based on actual bacterial concentrations of aerosol samples collected during the course of each exposure and plethysmography.

#### Body weight and post-exposure clinical monitoring

Animals were weighed every 7 days post-exposure as well as at termination (death, euthanasia, or scheduled study end). Each animal was observed for clinical signs a minimum of twice daily throughout the study and four times daily on days 1–21. Animals were followed for 45 days after exposure, at which point the experiment was scheduled to terminate. Animals experiencing hypothermia or displaying other humane endpoint criteria were anesthetized and euthanized.

#### Ceftobiprole medocaril and vehicle formulation

All antibiotic and formulation reagents were pharmaceutical grade. Ceftobiprole medocaril (Nipro Pharma Corporation; lot number 23K03) was reconstituted, diluted, and stored in infusion cassettes as described for phase I. Vehicle (5% dextrose [wt/vol] solution in water for injection; ICU Medical, Lake Forest, IL; lots 1006186 and 1010271) was obtained from a registered pharmacy.

#### Antibiotic and vehicle administration

Treatment for each animal was initiated 24 h after the time of fever onset (time at which fever was sustained for 2 h). Each animal received infusions of vehicle (delivered at a volumetric equivalent to ceftobiprole medocaril infusions, group 1) or ceftobiprole medocaril at one of the following doses: 1.33 mg/kg (infused as 0.2 mg/mL solution, group 2), 6.67 mg/kg (infused as 1.0 mg/mL solution, group 3), or 20.0 mg/kg (infused as 3.0 mg/mL solution, group 4), based on individual animal body weights measured on day 0. Based on predicted dissociation rates of the API, the nominal ceftobiprole doses were considered to be 1 mg/kg, 5 mg/kg, and 15 mg/kg, respectively. Infusions were 2 h in duration and were administered at intervals of 8 h based on the start time of the previous treatment. Once initiated, infusions were administered for 10 consecutive days (a total of 30 doses unless the animal died prior to completing treatments). Infusions were delivered to the jugular or femoral VAP as described above.

Blood samples for assessing plasma concentrations of BAL5788 and BAL9141 were collected and processed for LC-MS/MS as described above prior to infusion doses 1 and 10, as well as 2, 4, 6, and 8 h after the start of doses 1 and 10.

#### Model assessment

The presence of *F. tularensis* in whole blood was evaluated prior to the first treatment and on days 2, 4, 6, 8, 10, 12, 21, and 45 (or animal death) by serial dilution plating and qPCR to probe for a specific portion of *tul4* as previously described (21, 23, 24). Complete blood counts, differential WBC counts, and CRP levels were assessed prior to the first treatment and on days 2, 6, 8, 12, 21, and 45 as previously described (25). Serum samples derived from whole blood collected on days 4, 6, 10, and 45 (or animal death) were analyzed for the presence of anti-*F*. *tularensis* antibodies using a serum agglutination assay, as previously described (24).

Terminal bacterial burdens were determined for the lungs, liver, brain, meninges, spleen, mediastinal lymph nodes (MLN), and bone marrow for each animal by plating a sample on cystine heart agar with sheep blood and antibiotics, as previously described (23).

The minimum inhibitory concentrations (MICs) of ceftobiprole against *F. tularensis* isolates used for aerosol challenge on each day of exposure and for *F. tularensis* isolates from blood or tissues of animals that received at least one infusion of ceftobiprole medocaril were determined by broth macrodilution. The assay was performed as previously described (17), except that *F. tularensis* and *Staphylococcus aureus* isolates were normalized to a starting target concentration of 5.0 × 10^5^ CFUs/mL (range 2–8 × 10^5^ CFUs/mL) using a 0.5 McFarland Standard, Sensititre nephelometer model YV3011 (ThermoFisher Scientific, Waltham, MA), and dilution series. Furthermore, the antibiotic dilution series evaluated for each isolate consisted of 12 twofold dilutions ranging from 4 to 0.002 µg/mL ceftobiprole or 8 µg/mL–0.004 µg/mL ciprofloxacin.

### Statistical analysis (phase II)

#### Survival and time to death

Survival rates and exact 95% binomial confidence intervals were calculated for each treatment or control group. One-sided Boschloo’s tests were used to compare the survival rates between each treated group and the vehicle control group. Two-sided Boschloo’s tests were performed to compare survival rates between each pair of treated groups. The type I error rate was controlled at no more than 5% across the three treatment-to-control tests and at no more than 5% across the three pairwise tests of treated groups using the Bonferroni-Holm multiple comparison procedure.

Considering survival time as well as survival rates, a time-to-death analysis was performed to determine if there were differences in protection among the groups based on a length-of-survival model. Specifically, the time from challenge until death was calculated for each animal. Time to death for surviving animals was right-censored at the end of the study. Log-rank tests were used to determine if there were significant differences in times to death between the treated groups and the vehicle control group or between the pairwise treated groups. The type I error rate was controlled at no more than 5% across the treatment-to-control tests and at no more than 5% across the pairwise tests of treated groups using the Bonferroni-Holm multiple comparison procedure.

#### Body temperature

Post-challenge temperature data were adjusted to subtract each animal’s average temperature at the same hour of day in the pre-challenge baseline period. A mixed analysis of variance (ANOVA) model was fitted to the baseline-adjusted post-challenge data for each post-challenge day that included group as a fixed effect and animal as a random effect to account for the repeated measurements on the same animal over time. In this model, the least squares mean was estimated for each group, and a *t*-test was used to determine if there was a significant post-challenge shift from baseline for the group. A daily mean plot was generated for body temperature for the days after challenge to visually display analysis results. Pairwise *t*-tests from the model were used to determine if (i) the treated groups had mean shifts from baseline that were significantly different from that of the control animals; and (ii) there were mean shifts from baseline that were significantly different between each pair of treated groups. The type I e rror rate was controlled at no more than 5 % across the three treatment-to-control tests using Dunnett’s multiple comparison procedure and at no more than 5% across the three pairwise tests of the treated groups using Tukey’s multiple comparison procedure.

#### Ceftobiprole medocaril and ceftobiprole plasma concentrations

Values reported as below the limit of quantitation (BLQ) were replaced with one-half the limit of quantitation (10.0 ng/mL for ceftobiprole medocaril [BAL5788] and 50.0 ng/mL for ceftobiprole [BAL9141]). Values reported as no peak detected (ND) were replaced with zero for analysis; values reported as zero were replaced with one prior to log-transformation.

ANOVA models were fitted to the ceftobiprole medocaril and ceftobiprole plasma concentration data with fixed effects for group, time point, and the interaction between group and time point to assess normality and to identify potential outliers. A review of the results from the Shapiro-Wilk test as well as an examination of the normal probability plots of the residuals was used to assess normality. Deleted studentized residuals, which are the standardized residuals from the model fitted to all data except the current observation, were calculated for each observation. If the absolute value of the deleted studentized residual was greater than four, then the observation was considered an outlier. Mean levels and corresponding 95% confidence intervals were calculated with outliers excluded.

#### Blood and tissue burdens

Quantitative bacteremia and tissue burden data were base-10 log-transformed for analysis, with values of zero replaced with one prior to log-transformation. These data were summarized with geometric means and corresponding 95% confidence intervals by group, separately for each time point for the bacteremia data and for each tissue sample for the tissue burden data. An ANOVA model containing a fixed effect for group was fit to each log-transformed endpoint. Least square mean differences were estimated from this model, and *t*-tests were performed to determine if there were significant differences between the groups. The type I error rate was controlled at no more than 5% for the treated-to-control tests using Dunnett’s multiple comparison procedure, and no more than 5% for the tests between treated groups using Tukey’s multiple comparison procedure.

#### Body weight, hematology, and clinical chemistry

ANOVA models fitted separately to body weight and each hematology and clinical chemistry parameter with fixed effects for group, study day, and the interaction between group and study day were used to assess the model assumption of normality and to identify potential outliers. A review of the results from the Shapiro-Wilk test as well as an examination of the normal probability plots of the residuals was used to assess normality. Deleted studentized residuals were calculated for each observation. If the absolute value of the deleted studentized residual was greater than four, then the observation was considered a potential outlier.

ANOVA models were fitted to each endpoint/parameter, and least square means were estimated to determine if (i) there were significant changes from baseline for each post-challenge time point; (ii) there were statistically significant differences between the three treated groups and the untreated control group, with the type I error rate controlled at no more than 5% for the three tests using Dunnett’s multiple comparison procedure; and (iii) there were statistically significant differences between the three treated groups, with the type I error rate controlled at no more than 5% for the three tests using Tukey’s multiple comparison procedure. Baseline values were used to adjust for *a priori* differences between animals, with each animal serving as its own control.

## RESULTS

### Pharmacokinetic analysis (phase I)

All six CMs used in this phase completed each dosing round without adverse clinical observations or noteworthy changes in body weight. Clinical observations did not identify severe effects associated with any of the doses tested, with clinical observations limited to soft or absent feces (observed erratically and without association with dose administration).

#### Plasma concentrations of ceftobiprole medocaril and ceftobiprole

Each plasma sample was analyzed by LC-MS/MS to determine the concentration of BAL5788 (ceftobiprole medocaril) (Table S2) and BAL9141 (ceftobiprole) (Table S3). The LLOQ for the assay was 10.0 ng/mL (BAL5788) and 50.0 ng/mL (BAL9141), and the upper limit of quantification was 10,000 ng/mL (BAL5788) and 50,000 ng/mL (BAL9141). All analytical runs met acceptance criteria for the calibration standards and QC samples, and no carryover for BAL5788 or BAL9141 was detected.

#### Pharmacokinetic analysis

**Fig 1:**
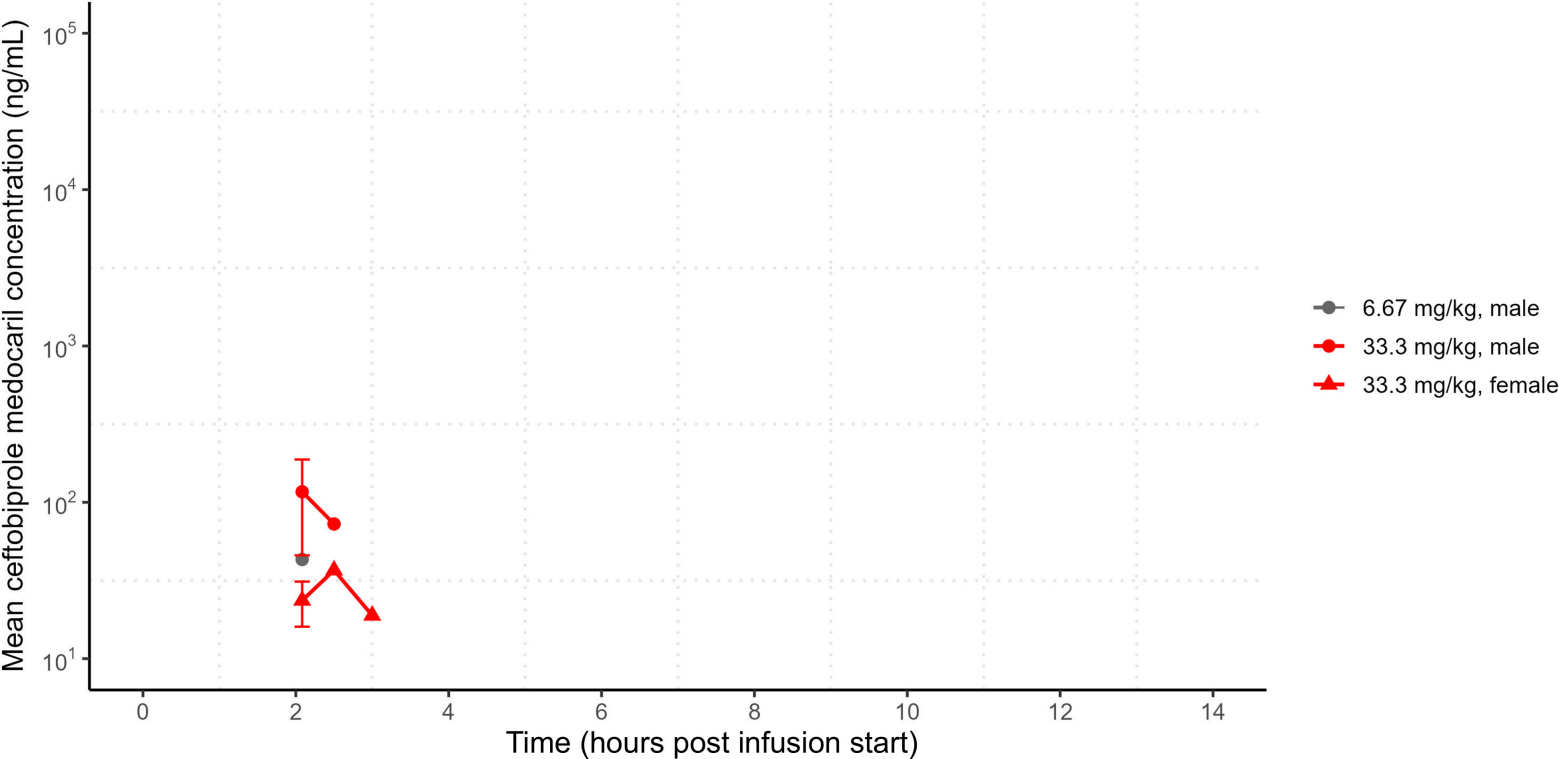
Male and female mean ceftobiprole medocaril plasma concentrations following single IV infusions. *n* = 3 per time point. Dosing regimen reported as ceftobiprole medocaril dosing regimen. t = 0 represents infusion start time, and t = 2 represents infusion stop time. PK parameters and statistical comparisons could not be determined because of the low rate of prodrug detection. Error bars represent standard deviation.

**Fig 2:**
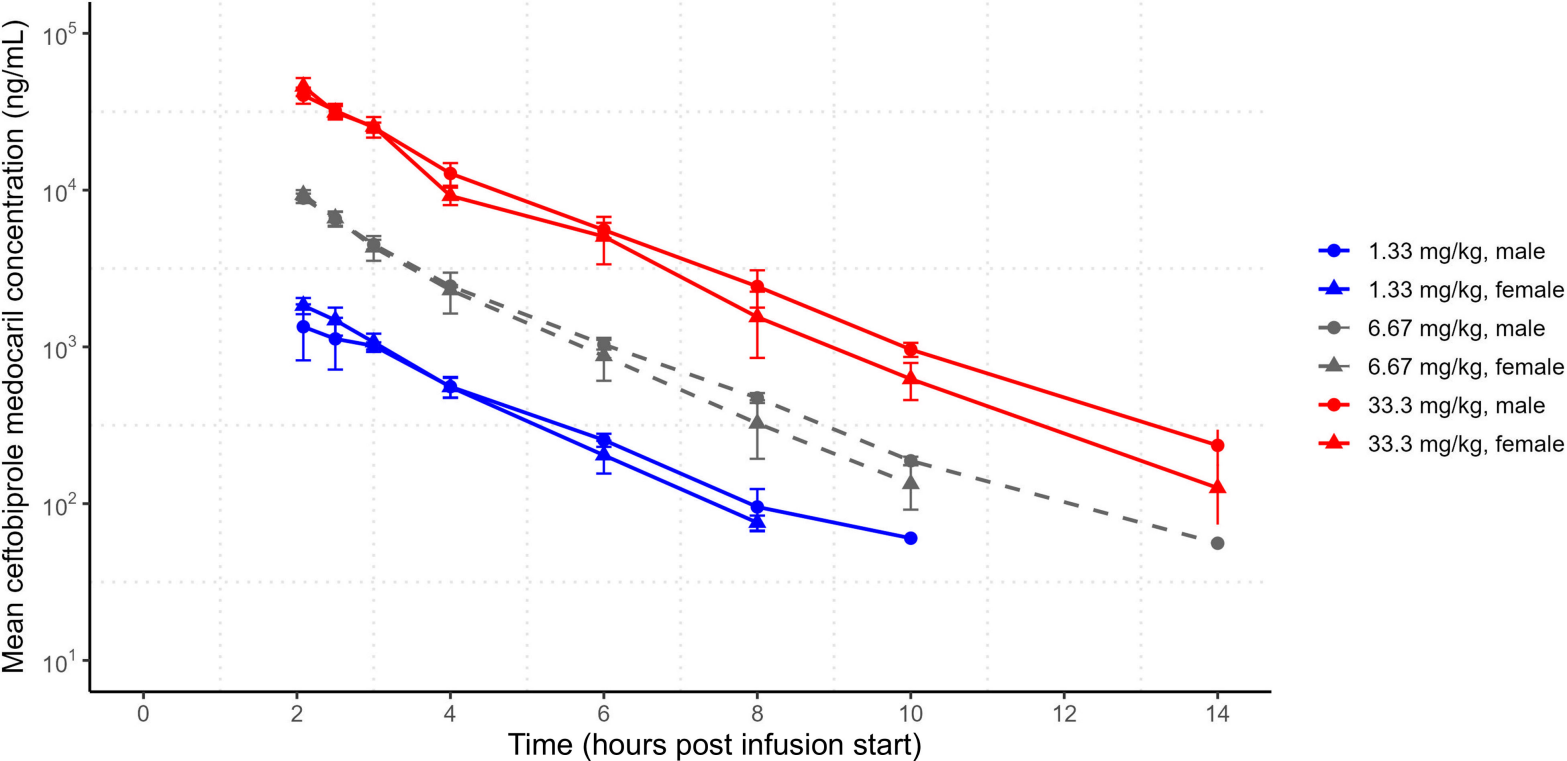
Male and female mean ceftobiprole plasma concentrations following single IV infusions. *n* = 3 per time point. Dosing regimen reported as ceftobiprole medocaril dosing regimen. t = 0 represents infusion start time, and t = 2 represents infusion stop time. Differences between the *C*_max_ and AUC_last_ of male and female CMs at each dose level were determined by unpaired *t*-test. C_max_ and AUC_last_ *P*-values at 1.33 mg/kg were 0.164 and 0.332, respectively; at 6.67 mg/kg were 0.442 and 0.787, respectively; and at 33.3 mg/kg were 0.280 and 0.789, respectively. Error bars represent standard deviation.

Low levels of ceftobiprole medocaril were detected 5 minutes after infusion stop times in six of six animals when administered at 33.3 mg/kg and one of six animals when administered at 6.67 mg/kg. Ceftobiprole medocaril was detected at subsequent time points only when infused at 33.3 mg/kg (two of six animals at 30 minutes and one of six animals at 1 h post-infusion stop). The ceftobiprole medocaril maximum concentration (*C*_max_) was observed at the end of infusion, and PK parameters could not be determined because the prodrug was rapidly converted to ceftobiprole upon infusion (Fig. 1). By comparison, ceftobiprole was detectable for up to 12 h post-treatment following infusions with 33.3 mg/kg or 6.67 mg/kg ceftobiprole medocaril and for up to 8 h post-treatment following the 1.33 mg/kg ceftobiprole medocaril infusions. The ceftobiprole *C*_max_ was also reached at the end of infusions. The exposures (*C*_max_ and area under the curve at the last measurable plasma concentration; *AUC*_*last*_) of ceftobiprole were approximately dose-proportional between 1.33 and 33.3 mg/kg ceftobiprole medocaril infusions, with low inter-individual variability and no significant differences between male and female CM exposures (Fig. 2). Each infusion dose resulted in a low volume of distribution (0.46 L/kg–0.50 L/kg) and a low plasma clearance (3.4 mL/min/kg–3.5 mL/min/kg) for ceftobiprole (Table 1), resulting in a half-life of around 1.6 h (the mean half-life of ceftobiprole in all animals [males and females] following infusions of 1.33, 6.67, or 33.3 mg/kg ceftobiprole medocaril was 1.46, 1.56, or 1.64 h, respectively).

**TABLE 1:** Mean or median plasma PK parameters of ceftobiprole after single IV infusions in cynomolgus macaques

Ceftobiprole medocaril dose	1.33 mg/kg	6.67 mg/kg	33.3 mg/kg
Median *T*_max_ (h) (minimum–maximum)	2.08 (2.08–2.5)	2.08 (2.08–2.08)	2.08 (2.08–2.08)
Mean *C*_max_ (ng/mL)	1,690 ± 248	9,110 ± 624	43,100 ± 5,730
Mean *C*_max_/dose ((ng/mL)/(mg/kg))	1,690 ± 248	1,820 ± 125	1,720 ± 229
Median *T*_last_ (h) (minimum–maximum)	8.00 (8.00–10.0)	10.0 (10.0–14.0)	14.0 (14.0–14.0)
Mean *C*_last_ (ng/mL)	75.5 ± 15.5	137 ± 53.2	181 ± 78.6
Mean AUC_last_ (h × ng/mL)	4,770 ± 712	24,200 ± 2,310	121,000 ± 11,600
Mean AUC_last_/dose ((h × ng/mL)/(mg/kg))	4,770 ± 712	4,850 ± 462	4,850 ± 463
Mean AUC_inf_ (h × ng/mL)	4,930 ± 721	24,500 ± 2,340	122,000 ± 11,700
Mean *T*_1/2_ (h)	1.46 ± 0.177	1.56 ± 0.148	1.64 ± 0.0940
Mean CL (mL/min/kg)	3.45 ± 0.570	3.42 ± 0.344	3.45 ± 0.327
Mean Vss (L/kg)	0.497 ± 0.111	0.457 ± 0.0295	0.477 ± 0.0529

### PK simulation and dose selection for phase II

**Fig 3:**
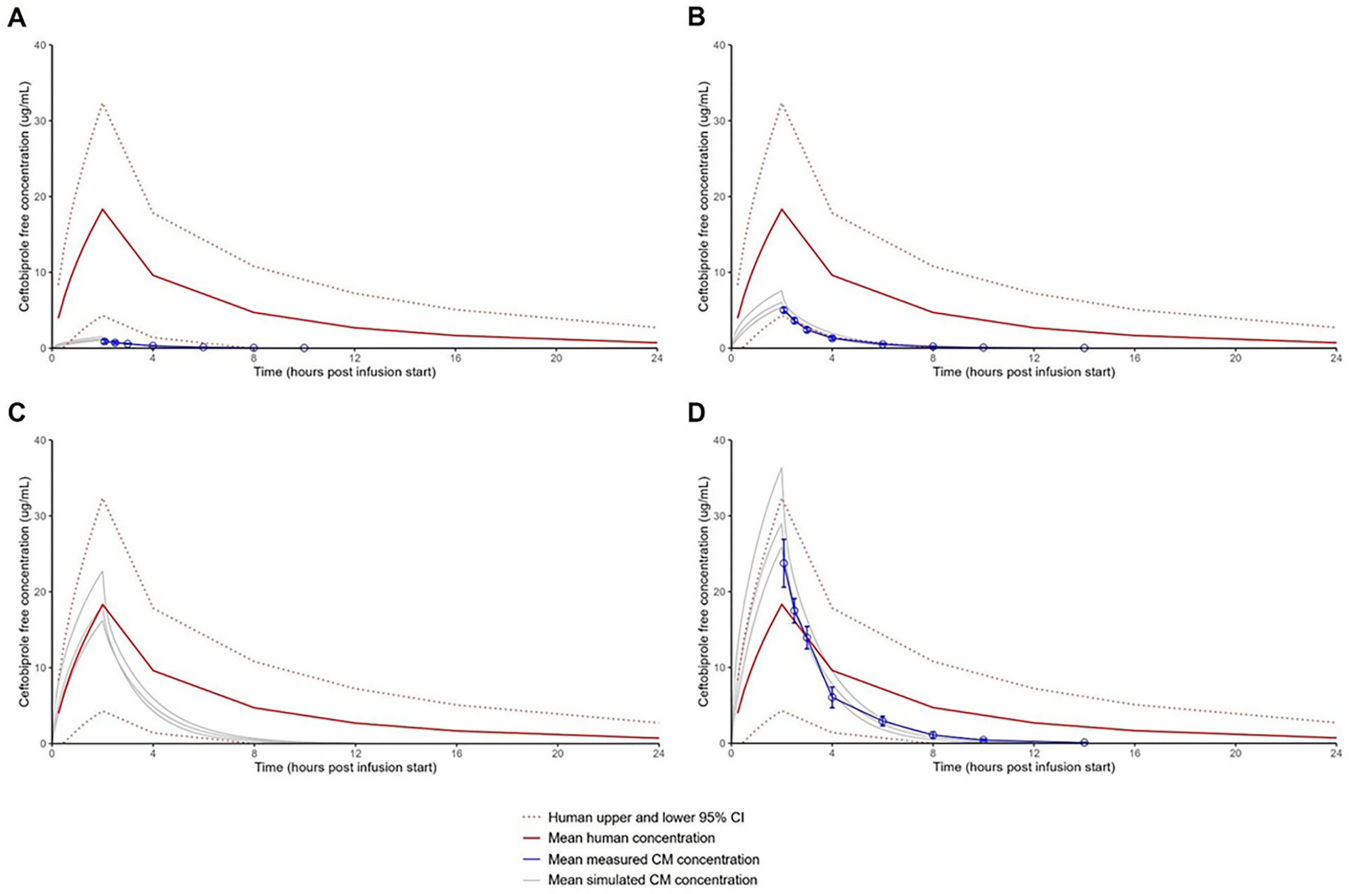
Simulated and measured plasma concentration-time profiles of ceftobiprole following a 2 h infusion. (**A–D**) Concentration-time profiles were simulated (*n* = 3) to predict the ceftobiprole exposures following one of four ceftobiprole medocaril doses (gray lines). (**C**) The concentration-time profile for 20.0 mg/kg ceftobiprole medocaril in CMs was simulated based on measured concentration-time profiles from (**A**) 1.33, (**B**) 6.67, and (**D**) 33.3 mg/kg ceftobiprole medocaril in CMs. Error bars on mean measured CM concentrations (blue symbols in panels **A**, **B, D**) represent standard deviation. The human mean concentration is also provided in each panel with the 95% confidence interval (CI).

The measured PK parameters were used to model and simulate PK concentration-time profiles for doses other than those administered in phase I (Fig. 3; Tables S4 and S5). Based on those findings, the three dose levels selected for phase II testing were 1.33 mg/kg, 6.67 mg/kg, and 20.0 mg/kg ceftobiprole medocaril (equivalent to 1, 5, and 15 mg/kg ceftobiprole), with the expectation that the 5 mg/kg ceftobiprole dose would provide a humanized dose equivalent to the lowest 95% confidence interval of the mean plasma concentration observed in humans (Fig. 3). The 1.33 mg/kg and 20.0 mg/kg ceftobiprole medocaril doses were selected to bracket this predicted humanized dose (i.e., 1.33 mg/kg, which would provide limited exposures, and 20.0 mg/kg, which would provide higher exposures).

### Therapeutic efficacy analysis (phase II)

#### Inhalational challenges

Animals were individually challenged with aerosolized *F. tularensis,* and the average inhaled exposure dose was 694 CFUs per animal (Table 2) with a range of 355–1,567 CFUs per animal (Table S6). Mass median aerodynamic diameter of exposure aerosols ranged from 1.19 to 1.43 µm for all animal exposures (geometric standard deviation of 1.78–1.93).

**TABLE 2:** Mean challenge doses by group, sex, and overall

Treatment or classification	Number of males	Number of females	Mean challenge dose (CFUs/animal)
Group 1	4	4	733
Group 2	5	3	612
Group 3	5	3	675
Group 4	4	4	755
Males	18	0	692
Females	0	14	696
All animals	18	14	694

#### Fever onset times, clinical observations, and body weights

All animals experienced a fever within 3.5 days of challenge, with an overall mean time from challenge to fever onset of 3.0 days (range 2.4–3.5 days) (Table 3). All animals were observed as clinically normal before inhalational exposures. In general, animals in each group initially displayed similar clinical signs beginning 3–5 days post-challenge. However, disease manifestation in controls tended to progress more consistently prior to termination. Although clinical signs of disease were observed in each of the groups treated with ceftobiprole medocaril, the frequency, day-to-day consistency, and severity of signs were generally limited compared to controls.

**TABLE 3:** Summary statistics for challenge to fever onset time (hours)

Group	Ceftobiprole medocaril dose (mg/kg)	*N*	Mean	Standard deviation	Median	Minimum	Maximum
1	0 (Vehicle control)	8	66.18	8.35	62.45	57.60	78.40
2	1.33	8	72.65	6.95	75.49	59.18	81.60
3	6.67	8	76.20	6.50	76.19	63.78	84.75
4	20.0	8	72.78	6.70	74.93	60.97	79.75

There were no significant differences in group mean body weights between any pair of groups prior to challenge with *F. tularensis*. Although animals in each group, on average, lost weight by days 14 and 21, there were no significant differences between the group mean body weights when comparing groups treated with ceftobiprole medocaril with the control group or with other treated groups on each day the animals were weighed post-challenge (Fig. S1).

#### Survival and time to death

**Fig 4:**
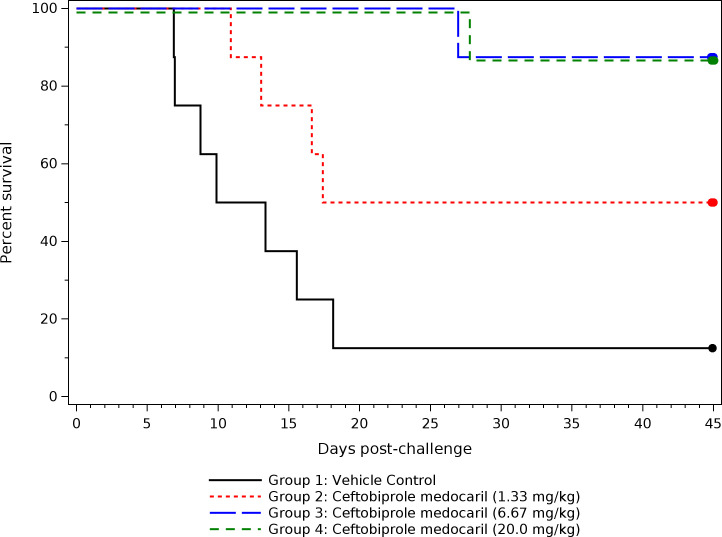
Survival of CMs receiving placebo or antibiotic treatments. Kaplan-Meier curves were generated based on the challenge time and time to death of individual animals. Group 1 survival rate was 12.5% (95% confidence interval: 0%–53%). Group 2 survival rate was 50% (95% confidence interval: 16%–84%). Group 3 and group 4 survival rates were 87.5% (95% confidence interval: 47%–100%). Survival rate for groups 3 and 4 was slightly offset to distinguish between the groups. The solid dots indicate scheduled study termination.

Among the group 1 animals (receiving vehicle infusions), there was a 12.5% survival rate (Fig. 4). The survival rates among groups of animals that received treatments with ceftobiprole medocaril ranged from 50% (group 2) to 87.5% (groups 3 and 4). Although the 50% survival rate among group 2 animals was not statistically significantly different from group 1 (*P* = 0.0682) or from groups 3 and 4 (*P* = 0.4089), treatment with 6.67 mg/kg or 20.0 mg/kg ceftobiprole medocaril (groups 3 and 4, respectively) was associated with a statistically significant difference in survival rates compared to the group 1 control animals (*P* = 0.0063). No statistical differences in survival were evident between groups 3 and 4 (*P* = 1.0000). Differences in median time to death among CMs in each group followed a similar pattern (Table 4). Although the median and upper confidence bounds could not be estimated for the treatment groups due to the high survival rates, times to death were significantly longer for groups 3 and 4 compared to group 1 (11.63 days post-challenge, *P* = 0.0025). No other significant differences in times to death between any other groups were identified.

**TABLE 4:** Median times to death of CMs receiving placebo or antibiotic treatments with 95% confidence intervals by group

Group	Ceftobiprole medocaril dose (mg/kg)	No. died/no. in group	Kaplan-Meier median time to death in days (95% confidence interval)	*P*-value for comparison between treated groups and control	*P*-value for comparison among treated groups	
				Group 1	Group 3	Group 4
1	0 (Vehicle control)	7/8	11.63 (6.89, 18.13)	NA^*c*^	NA	NA
2	1.33	4/8	-- (10.90, --)^*b*^	0.0652	0.2560	0.2560
3	6.67	1/8	-- (26.97, --)^*b*^	0.0025^*a*^	NA	0.9624
4	20.0	1/8	-- (27.78, --)^*b*^	0.0025^*a*^	NA	NA

^*a*^ Time to death was significantly greater for the treated group than for the control group.

^*b*^ Median and upper confidence bound could not be estimated due to high survival rate.

^*c*^ NA, not applicable.

#### Detection of F. tularensis in animals post-challenge

Of the samples collected from animals treated with ceftobiprole medocaril (groups 2–4) for quantitative bacteremia analysis, none resulted in identification of *F. tularensis* at a burden greater than the LLOQ (250 CFUs/mL) (Table 5). Among the group 1 animals, quantitative analysis identified *F. tularensis* in blood samples as early as 2 days post-challenge and as late as 12 days post-challenge, though detection was generally sporadic, as the individual animals with positive results varied longitudinally (data not shown).

**TABLE 5:** Proportion of animals with positive quantitative bacteremia and mean burden of positive samples

Group	1		2	3	4
Ceftobiprole medocaril dose	0 (Vehicle control)		1.33 mg/kg	6.67 mg/kg	20.0 mg/kg
Sample collection	No. > LLOQ/*N*	Mean burden (CFUs/mL) of positive samples	No. > LLOQ/*N*		
Prior to treatment	0/8	NA	0/8	0/8	0/8
2 days post-challenge	1/8	1.34 × 10^3^	0/8	0/8	0/8
4 days post-challenge	0/8	NA	0/8	0/8	0/8
6 days post-challenge	1/8	4.40 × 10^2^	0/8	0/8	0/8
8 days post-challenge	0/6	NA	0/8	0/8	0/8
10 days post-challenge	1/5	4.53 × 10^4^	0/8	0/8	0/8
12 days post-challenge	1/4	2.70 × 10^3^	0/7	0/8	0/8
21 days post-challenge	0/1	NA	0/4	0/8	0/8
45 days post-challenge	0/1	NA	0/4	0/7	0/7
Terminal	2/5	4.12 × 10^3^	0/3	0/1	0/0^*a*^

^*a*^ A sample was unable to be collected for the single terminal observation.

Quantitative PCR was performed to aid in the detection of *F. tularensis* DNA in blood. *F. tularensis* DNA was detected as early as 2 days post-challenge and confirmed the presence of *F. tularensis* DNA in all animals on at least 1 day post-challenge (Table 6).

**TABLE 6:** Proportion of animals with positive qPCR result

Group	1	2	3	4
Ceftobiprole medocaril dose	0 (Vehicle control)	1.33 mg/kg	6.67 mg/kg	20.0 mg/kg
Sample collection	No. qPCR^+^/*N*			
Prior to treatment	5/8	6/8	5/8	6/8
2 days post-challenge	0/8	0/8	1/8	0/8
4 days post-challenge	7/8	5/8	5/8	6/8
6 days post-challenge	8/8	8/8	7/8	7/8
8 days post-challenge	6/6	8/8	7/8	8/8
10 days post-challenge	5/5	8/8	6/8	8/8
12 days post-challenge	4/4	6/7	8/8	4/8
21 days post-challenge	0/1	2/4	4/8	3/8
45 days post-challenge	0/1	0/4	1/7	1/7
Terminal	6/6^*a*^	3/3^*a*^	1/1	0/0^*a*^

^*a*^ A sample was unable to be collected from a terminal animal.

#### Analysis of host factors in blood

Changes in host factors relative to baseline (measured 14 days before *F. tularensis* challenge), such as WBC counts, neutrophil counts, lymphocyte counts, and CRP, were most evident prior to treatment (Fig. S2 to S5). Average WBC counts were elevated above the Battelle clinical reference range prior to treatment in groups 1 and 4, and neutrophil counts were elevated above the reference range in all groups, though group averages returned to reference ranges at post-treatment time points. CRP elevation was evident prior to treatment in all groups and remained above the reference range for most of the post-challenge follow-up period. Group mean CRP levels returned to the reference range by day 45 post-challenge only in groups treated with ceftobiprole medocaril.

An adaptive immune response to *F. tularensis* (assessed through detection of anti-*F*. *tularensis* antibodies by serum agglutination assay) was confirmed in all animals that survived to day 45 post-challenge (Table 7). All animals were negative for *F. tularensis* antibody agglutination prior to *F. tularensis* exposure. The earliest positive results for anti-*F*. *tularensis* antibodies were detected 10 days post-challenge, with evidence of an immune response represented in all groups. Furthermore, there were four control animals (group 1) and two treated with 1.33 mg/kg ceftobiprole medocaril (group 2) that never tested positive for *F. tularensis* antibody agglutination, though each of these animals died prior to the end of study, and infection was confirmed in each animal by CFU recovery from tissues (described below).

**TABLE 7:** Proportion of animals with positive agglutination result for anti-*F. tularensis* antibodies

Group	1	2	3	4
Ceftobiprole medocaril dose	0 (Vehicle control)	1.33 mg/kg	6.67 mg/kg	20.0 mg/kg
Sample collection	No. seropositive/*N*			
4 days post-challenge	0/8	0/8	0/8	0/8
6 days post-challenge	0/8	0/8	0/8	0/8
10 days post-challenge	2/5	4/8	7/8	7/8
45 days post-challenge	1/1	4/4	7/7	7/7
Terminal	3/6^*a*^	2/3^*a*^	1/1	0/0^*a*^

^*a*^ A sample was unable to be collected from a terminal animal.

#### Bacterial tissue burdens

**Fig 5:**
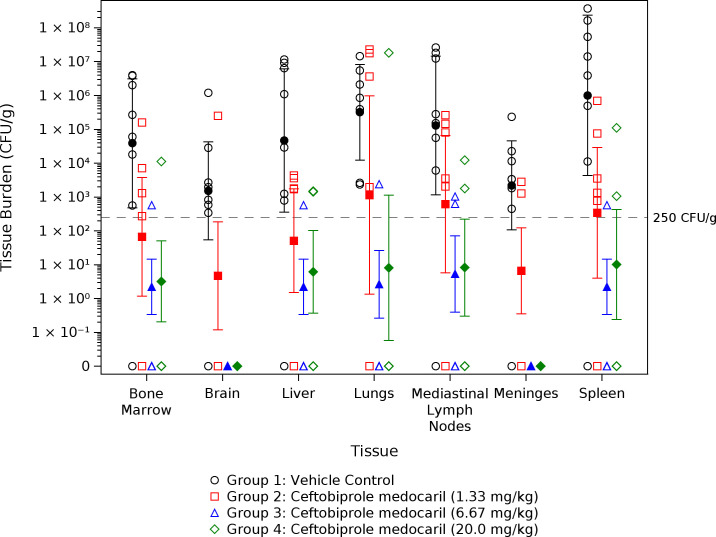
Geometric mean bacterial tissue burdens. Solid markers represent group geometric means with 95% confidence intervals. For each tissue type, the limit of quantification was < 250 CFUs/g (dashed gray line).

Of the seven control animals (group 1) that died before day 45 post-challenge, *F. tularensis* was detected in all tissue samples analyzed (Fig. 5). Bacterial concentrations were >LLOQ (250 CFUs/g) in ~82% (40/49) of samples. However, *F. tularensis* was not detected in any tissue sample collected from the sole surviving animal in group 1 (Fig. 5). Among the animals treated with ceftobiprole medocaril, the highest proportion of animals with *F. tularensis* detected in at least one tissue sample occurred in group 2 animals (five of eight NHPs had *F. tularensis* present in tissues). By comparison, tissues from two of eight group 3 NHPs or two of eight group 4 NHPs were positive for *F. tularensis* (including the two animals that did not survive to 45 days post-challenge as well as two survivors). Among these four animals, half of the tissue samples (14/28) were positive for *F. tularensis,* though no evidence of infection was detected in brains or meninges (Fig. 5). The geometric mean tissue burdens for vehicle-treated animals (group 1) were significantly greater than those of each group treated with ceftobiprole medocaril for bone marrow, brain, liver, meninges, and spleen (Table 8). Lung and MLN geometric mean burdens of group 1 animals were also significantly greater than those of animals receiving 6.67 or 20.0 mg/kg ceftobiprole medocaril infusions (Table 8). No statistical pairwise differences in geometric mean tissue burdens were identified between groups treated with various ceftobiprole medocaril doses (Table 8).

**TABLE 8:** ANOVA results for comparisons of tissue burdens between groups

Tissue sample	Significant comparisons for treated vs control^*a*^	Significant comparisons among treated groups
Bone marrow	578.55 (1 > 2) 0.0115 17,482.67 (1 > 3) 0.0001 12,060.38 (1 > 4) 0.0002	NS^*b*^
Brain	322.89 (1 > 2) 0.0016 1,528.79 (1 > 3) <0.0001 1,528.79 (1 > 4) <0.0001	NS
Liver	905.33 (1 > 2) 0.0073 20,963.20 (1 > 3) 0.0001 7,503.74 (1 > 4) 0.0005	NS
Lungs	120,376.11 (1 > 3) 0.0010 39,415.22 (1 > 4) 0.0027	NS
MLN	24,561.90 (1 > 3) 0.0005 15,829.31 (1 > 4) 0.0009	NS
Meninges	336.82 (1 > 2) 0.0002 2,216.06 (1 > 3) <0.0001 2,216.06 (1 > 4) <0.0001	NS
Spleen	2,938.58 (1 > 2) 0.0080 455,323.28 (1 > 3) <0.0001 98,745.02 (1 > 4) 0.0002	NS

^*a*^ Cells contain pairwise differences that were statistically significant at the 0.05 level. The format within each cell is (i) the ratio of geometric means between groups, (ii) the relationship between the corresponding pair of group geometric means shown in parentheses (for example, “(1 > 2)” indicates that the geometric mean of group 1 was greater than that of group 2), and (iii) the Dunnett-adjusted *P*-value for the treated to control test.

^*b*^ NS, no significant comparisons.

#### Minimum inhibitory concentration

In addition to the two isolates used on each inhalational challenge day, a total of 45 *F*. *tularensis* isolates from blood or tissue samples of NHPs that had received at least one ceftobiprole medocaril treatment were tested for MICs of ceftobiprole and ciprofloxacin (as a control article). Isolates were evaluated over a series of four assays, with each assay exhibiting passing controls (growth in positive control growth tubes and no evidence of contamination in negative control samples). *S. aureus* was also included in each assay as a QC organism and was evaluated against ceftobiprole and ciprofloxacin. The MICs of ceftobiprole and ciprofloxacin against *S. aureus* in each assay (0.25 µg/mL–0.5 µg/mL and 1.0 µg/mL, respectively) were on scale with previous results using the broth macrodilution method (17) (ceftobiprole and ciprofloxacin) and the CLSI reference range (ciprofloxacin only and for cation-adjusted Muller-Hinton broth with 2% IsoVitalex) (26).

All samples were evaluated at 24 and 48 h, although no differences in the MICs of ceftobiprole or ciprofloxacin against a specific isolate were detected between the two endpoints. The two isolates used for inhalational challenges had equivalent MICs of ceftobiprole (0.06 µg/mL) or ciprofloxacin (0.015 µg/mL) (Table 9). All 45 isolates demonstrated a ceftobiprole MIC equal to or less than the isolates used for inhalational challenges (range of 0.008 µg/mL–0.06 µg/mL) (Table 9). Although 10 isolates had a ciprofloxacin MIC greater than the challenge material isolates (0.03 µg/mL–0.06 µg/mL), these differences were within a three-dilution range and thus likely due to an expected level of variation in the assay, as ciprofloxacin was not administered to NHPs (27).

**TABLE 9:** Minimum inhibitory concentration of ceftobiprole against *F. tularensis* isolates

Sample	Organism	Number of isolates evaluated	Ceftobiprole MIC range (µg/mL)	Ciprofloxacin MIC (µg/mL)
Quality control organism	*S. aureus*	4	0.25–0.5	1.0
Challenge isolate 1	*F. tularensis*	1	0.06	0.015
Challenge isolate 2	*F. tularensis*	1	0.06	0.015

*F. tularensis* samples isolated from	Number of isolates tested	Tissues represented	Ceftobiprole MIC range (µg/mL)	Ciprofloxacin MIC range (µg/mL)
Group 2 NHPs	29	Lung, liver, brain, meninges, spleen, MLN, bone marrow, blood	0.008–0.06	0.008–0.06
Group 3 NHPs	6	Lung, liver, spleen, MLN, bone marrow	0.03–0.06	0.015
Group 4 NHPs	10	Lung, liver, spleen, MLN, bone marrow, blood	0.008–0.06	0.015–0.03

#### Ceftobiprole medocaril and ceftobiprole exposures

**Fig 6:**
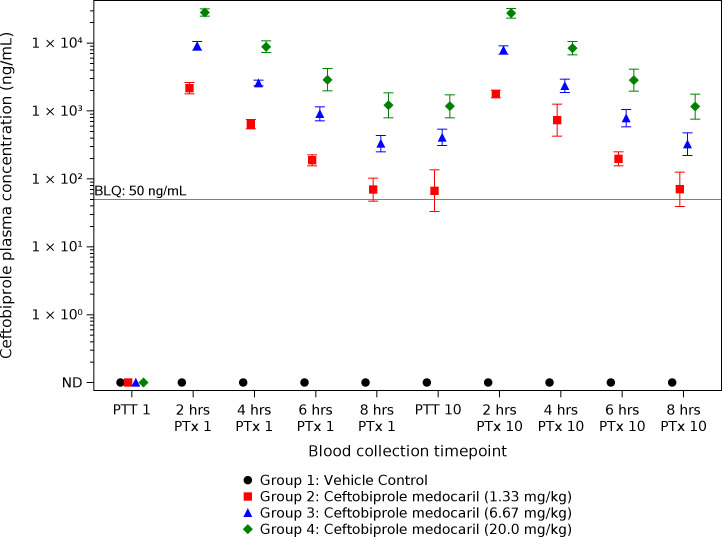
Geometric mean ceftobiprole plasma concentration versus time in challenged CMs. BLQ: Below the limit of quantification; ND: not detected; PTT 1, prior to treatment number 1; PTT 10, prior to treatment number 10; PTx 1, post-treatment number 1; PTx 10, post-treatment number 10. Group markers at each time point are slightly offset to distinguish between the groups (*x*-axis does not represent a time scale). Error bars represent 95% confidence interval.

Plasma concentrations of ceftobiprole medocaril and ceftobiprole were determined before and after doses 1 and 10. All LC-MS/MS analytical runs met acceptance criteria for both ceftobiprole medocaril and ceftobiprole calibration standards, QC precision and accuracy samples, and carryover between runs. Similar to PK analysis, ceftobiprole medocaril was rarely detectable in plasma samples (Fig. S6). Ceftobiprole was not detected in vehicle control samples (as expected) and was detected in a majority of samples collected after the initial treatment. The concentrations in each group were generally highest at the 2 h post-treatment time points and decreased as time since the treatment progressed (Fig. 6). Furthermore, the concentrations of ceftobiprole detected at each time point tended to correlate with the treatment dose, with group 4 samples having the highest concentrations compared to group 3 and group 2. For NHPs treated with ≥6.67 mg/kg ceftobiprole medocaril (group 3 and group 4), geometric mean ceftobiprole plasma concentrations at each interval remained greater than the highest MIC detected (0.06 µg/mL).

## DISCUSSION

Multiple animal models have been developed for researching tularemia and for the development and testing of potential treatments against the disease (16, 19, 21). A previous study testing ceftobiprole medocaril IV infusions in Fischer 344 rats demonstrated that this therapy is effective at a dosing regimen that mimicked the exposure of the human clinical dosing regimen (17). This pilot study using a small animal model provided justification for further development and testing of ceftobiprole medocaril for use against inhalational tularemia in a large animal model. Our approach was optimized by applying the FDA-qualified model of inhalational tularemia in CMs and represents the first use of ceftobiprole medocaril in a large animal model of inhalational *F. tularensis* infections.

Prior to evaluating the efficacy of ceftobiprole medocaril IV infusions against inhalational tularemia, PK analysis of ceftobiprole medocaril and ceftobiprole following infusions with potential dose levels was needed to establish PK parameters such as *C*_max_, AUC_last_, and the half-life in CMs. Infusions of ceftobiprole medocaril were well-tolerated. The overall low quantification levels and rapidly diminishing rate of ceftobiprole medocaril detection as time from infusions elapsed demonstrated that, upon infusion, the prodrug was rapidly converted to API (as expected [28, 29]), and that both the ceftobiprole medocaril and ceftobiprole *C*_max_ were reached at the end of infusions due to the rapid rate of conversion. Robust detection of ceftobiprole at all dose levels for at least 8 h after infusion stop times and up to 12 h after infusion stop times (the last time point assessed) provides support that the exposures (*C*_max_ and AUC_last_) of ceftobiprole are dose-proportional and consistent between male and female CMs. Furthermore, each dose level tested yielded similar half-life results, suggesting that the ceftobiprole half-life in CMs is relatively short (1.6 h). These data, along with modeling of additional doses and PK concentration-time profiles in humans, indicated that the 6.67 mg/kg ceftobiprole medocaril dose was likely to provide effective treatment against inhalational tularemia, and that the highest dose tested (33.3 mg/kg ceftobiprole medocaril) would likely exceed the human clinical exposures, thus providing little value if later demonstrated to be effective against inhalational tularemia in the CM model. Therefore, an intermediate dose (20.0 mg/kg ceftobiprole medocaril) was selected as the upper end for testing in the phase II efficacy experiment, as this dose would still bracket the predicted effective dose (6.67 mg/kg ceftobiprole medocaril) and had the potential to show a dose-response relationship (in terms of efficacy) while still representing human clinical exposures.

The inhalational exposure dose of *F. tularensis* is a critical factor in the CM model of tularemia (30). The target challenge dose in phase II (1,000 CFUs per animal) was selected based on the model range of 300–3,000 CFUs, which is considered statistically equivalent (19). All actual challenge doses for individual NHPs occurred within this range, and the average exposures of animals on each day of exposure, dose groups, or sex cohorts demonstrated minimal variation, and thus, the exposures were appropriate for the experimental objectives. Another critical factor for inhalation models is the characteristics of the aerosols. The MMAD and ranges of particle sizes detected throughout challenge runs were consistent between runs and were within the respirable range required for deposition of *F. tularensis* particles in the alveolar region of the lower respiratory tract and thus appropriate for the inhalational disease model (30).

Following exposure to *F. tularensis*, all NHPs demonstrated a fever response within 3.5 days of challenge, in line with previous applications of this disease model (22–25), and confirmation of fever in individual NHPs was used to determine treatment initiation times. After the initial period of fever, animals receiving a placebo infusion had a high rate of NHPs experiencing hypothermia, resulting in a median time to death of 11.63 days and an overall survival rate of 12.5%. Although the survival rate of NHPs receiving the lowest dose was limited (50%), each of the groups treated with higher ceftobiprole medocaril doses (6.67 or 20.0 mg/kg) demonstrated a statistically significant difference from the control group, with an 87.5% survival rate. Furthermore, for each NHP in the higher-dose groups that died (one per group), the times to death were longer than the median time to death and upper 95% confidence interval of control animals. The times of death for these two NHPs also occurred after antibiotic treatments had ended, suggesting death from latent infections that may have been initially suppressed but not fully eradicated by the 10 day treatment regimens (as opposed to ceftobiprole medocaril not being effective against *F. tularensis*). Although a dose-response relationship between the two higher-dose treatment groups was predicted by PK modeling, with only one death in each group, no statistical differences in survival or median time to death could be identified.

During the onset of disease, clinical observations were generally similar between groups, and initial signs tended to coincide with quantitative metrics, such as confirmation of fever. However, as days from fever confirmation progressed, control animals demonstrated disease progression more evidently than those in the higher-dose treatment groups. Consistent with human tularemia, all animals experienced decreases in lymphocyte counts and increases in neutrophil counts and CRP prior to treatment. Furthermore, CRP values returned to normal clinical ranges for most surviving animals, suggesting a positive clinical progression at 45 days post-challenge. Although detection of *F. tularensis* in blood by culture was sporadic (with quantifiable bacteremia limited to control animals, predominantly in terminal samples), secondary confirmation of *F. tularensis* infection included qPCR analysis, which confirmed the presence of *F. tularensis* genetic material in all animals post-challenge, including samples collected from surviving animals at earlier time points. Serum agglutination was also conducted to determine if an adaptive immune response was evident from NHPs infected with *F. tularensis*. Because all samples were non-reactive to *F. tularensis* antibody agglutination prior to exposure, the animals were considered naïve to *F. tularensis,* and thus, the detection of agglutinating antibodies in samples collected post-challenge indicates seroconversion among NHPs. This response was evident in most NHPs, including all survivors, with the few that did not demonstrate seroconversion succumbing to disease at earlier time points, likely before adaptive immunity could develop.

Analysis of terminal tissue burdens did identify statistically significant differences between groups. *F. tularensis* was detected in all tissues of placebo control NHPs that died during the study, and the bacterial tissue burdens were significantly greater than most mean tissue burdens of NHPs treated with 1.33 mg/kg ceftobiprole medocaril or all mean tissue burdens of NHPs treated with ≥6.67 mg/kg ceftobiprole medocaril. These data indicate that the moribund conditions and deaths of control animals were in fact due to disseminated *F. tularensis* infection. The lack of *F. tularensis* identified in the sole control NHP that survived is consistent with this conclusion, as the animal appeared to naturally counteract the infection and developed evidence of adaptive immunity. Among individual NHPs that were treated with ≥6.67 mg/kg ceftobiprole medocaril and survived, tissue burdens were either negative or low, suggesting that treatment with either dose of ceftobiprole medocaril may have supported clearance of the infection in conjunction with an adaptive immune response. Notwithstanding, the two NHPs that died after antibiotic treatment cessation (6.67 or 20.0 mg/kg ceftobiprole medocaril) had *F. tularensis* detected in five of seven tissue types evaluated (with brain and meninges remaining sterile), providing further support that deaths were due to resurgence of latent infections. Although the mutational frequency of *F. tularensis* following ceftobiprole medocaril exposure could be informative in the developmental pathway, ceftobiprole plasma concentrations among NHPs treated with ≥6.67 mg/kg ceftobiprole medocaril and the stability of MICs measured from isolates derived from these animals (relative to challenge isolate MIC) did not suggest development of resistance, and thus, mutational frequency was not assessed. Additionally, the presence of *F. tularensis* in two surviving animals suggests that additional resurgence or deaths could have been possible if animals were monitored for a longer period post-challenge. Together, these data suggest that prolonged treatment durations at the dosing regimens tested may be necessary for the use of ceftobiprole medocaril against inhalational tularemia, as sterilizing outcomes have been established in other developmental uses of this model with 14 day treatment periods (23).

Finally, the concentration of ceftobiprole achieved in the plasma is another critical factor in determining clinical efficacy against *F. tularensis*. Plasma concentrations were assessed relative to initial dosing (dose 1) and after dose 10. As expected, there was minimal detection of the prodrug (ceftobiprole medocaril) due to its rapid conversion to ceftobiprole, as established in phase I of this study. Consistent with phase I PK analysis, concentrations of ceftobiprole in each treatment group were highest immediately after infusions were completed and decreased steadily as time from infusion increased. A dose-response relationship was also evident, as samples from NHPs treated with 20.0 mg/kg ceftobiprole medocaril tended to have the highest concentrations compared to those of NHPs treated with 6.67 mg/kg and 1.33 mg/kg ceftobiprole medocaril, respectively. The plasma concentrations achieved from treatments with ≥6.67 mg/kg ceftobiprole medocaril are likely appropriate for supporting infection clearance, as CFUs from tissues were diminished and no increases in the MICs of isolates from these animals were detected compared to the MIC of *F. tularensis* isolates used for exposures.

### Conclusion

Overall, the PK analysis successfully predicted two dosing regimens that model/bracket human clinical exposures and preliminarily demonstrated efficacy against *F. tularensis* in the CM model of tularemia. The results from the challenge phase of this study consistently demonstrated that inhalational exposure to *F. tularensis* causes a highly lethal infection that represents human tularemia. Although a significant improvement in survival was not evident from the lowest dose treatment evaluated, treatment with ceftobiprole medocaril at 6.67 or 20.0 mg/kg administered by IV infusions may be an effective treatment or contribute to significant reductions in bacterial burdens. More research to optimize the survival of animals receiving similar dosing regimens or demonstrate complete clearance of bacteria from tissues will be necessary for further development of ceftobiprole medocaril as a treatment for tularemia. Building on the initial dosing regimen tested, and considering the upper limit of human clinical exposures and the dose-response relationship established herein, survival and tissue clearance as a result of prolonged treatments (>10 days) with 6.67 mg/kg–20.0 mg/kg ceftobiprole medocaril provide a promising developmental pathway. Finally, establishing an optimized treatment regimen in terms of dose concentration, infusion duration, and total treatment length will allow for enhanced evaluation of ceftobiprole medocaril, such as determining its treatment efficacy when administered at a time greater than 24 h post-fever confirmation and thus potentially increasing its clinical relevance for the treatment of tularemia.

## SUPPLEMENTAL MATERIAL

Supplemental Material aac.01278-25-s0001.docx Supplemental tables and figures.

ASM does not own the copyrights to Supplemental Material that may be linked to, or accessed through, an article. The authors have granted ASM a non-exclusive, world-wide license to publish the Supplemental Material files. Please contact the corresponding author directly for reuse.
