# Supplementary material for: Pharmacokinetic modeling and efficacy of ceftobiprole medocaril against pneumonic tularemia in cynomolgus macaques
Source: Antimicrob Agents Chemother. 2025 Nov 18;69(12):e01278-25. doi: 10.1128/aac.01278-25 (PMC12691666; doi:10.1128/aac.01278-25)
Supplement: Supplemental Material — Supplemental tables and figures. [file aac.01278-25-s0001.docx]

**Table S1. Chromatographic system settings.**

| Mass Spectrometer | AB SCIEX Qtrap 6500 with Turbo Ion Spray probe | | | | | | | | | | |
| --- | --- | --- | --- | --- | --- | --- | --- | --- | --- | --- | --- |
| HPLC | Shimadzu Prominence XR Series | | | | | | | | | | |
| Data Acquisition Software | Analyst 1.7.1 | | | | | | | | | | |
| Column | Thermo Fisher Scientific Hypersil Gold, 50 x 2.1 mm, 3 µm | | | | | | | | | | |
| Column Temperature | 30°C | | | | | | | | | | |
| Mobile Phase | A: 1% Formic Acid in Water | | | | | | | | | | |
|  | B: 1% Formic Acid in 50:50 Acetonitrile:Methanol | | | | | | | | | | |
| Time Program | Time (min) | | Flow Rate (mL/min) | | | %A | | | %B | | |
|  | 0.01 | | 0.500 | | | 100 | | | 0 | | |
|  | 0.50 | | 0.500 | | | 100 | | | 0 | | |
|  | 3.00 | | 0.500 | | | 50 | | | 50 | | |
|  | 3.10 | | 0.500 | | | 5 | | | 95 | | |
|  | 4.00 | | 0.500 | | | 5 | | | 95 | | |
|  | 4.10 | | 0.500 | | | 100 | | | 0 | | |
|  | 5.00 | | 0.500 | | | 100 | | | 0 | | |
| Flow Rate | 0.500 mL/min | | | | | | | | | | |
| Injection Volume | 5 µL | | | | | | | | | | |
| Needle Wash | 1:1:1 Acetonitrile:Methanol:2-propanol | | | | | | | | | | |
| Autosampler Temperature | 8°C | | | | | | | | | | |
| Acquisition Time | 5 minutes | | | | | | | | | | |
| Scan Type | MRM | | | | | | | | | | |
| Polarity | Positive | | | | | | | | | | |
| Ion Source | TurboIonSpray | | | | | | | | | | |
| CUR | 20 | | | | | | | | | | |
| CAD | 8 (Medium) | | | | | | | | | | |
| IS | 5500 | | | | | | | | | | |
| TEM | 500 | | | | | | | | | | |
| GS1 | 50 | | | | | | | | | | |
| GS2 | 50 | | | | | | | | | | |
| Transition Ions | Analyte | Q1 Mass (Da) | | Q3 Mass (Da) | Dwell (msec) | | DP (Volts) | EP (Volts) | | CE (Volts) | CXP (Volts) |
|  | BAL9141 | 535.1 | | 203.0 | 250 | | 54 | 10 | | 28 | 9 |
|  | BAL9141-d_4_ | 539.1 | | 207.0 | 250 | | 54 | 10 | | 28 | 9 |
|  | BAL5788 | 691.1 | | 535.1 | 250 | | 54 | 10 | | 18 | 9 |
|  | BAL5788-d_4_ | 695.1 | | 539.1 | 250 | | 54 | 10 | | 18 | 9 |

**Table S2. Plasma concentrations of ceftobiprole medocaril (BAL5788).**

| **Animal ID** | **Sex** | **Ceftobiprole medocaril Dose (mg/kg)** | **Determined Concentrations (ng/mL)** | | | | | | | | |
| --- | --- | --- | --- | --- | --- | --- | --- | --- | --- | --- | --- |
|  |  |  | **PTT** | **5 min PTx** | **30 min PTx** | **1 hr PTx** | **2 hrs PTx** | **4 hrs PTx** | **6 hrs PTx** | **8 hrs PTx** | **12 hrs PTx** |
| 1 | Male | 1.33 | ND | ND | ND | ND | ND | ND | ND | ND | ND |
| 2 |  |  | ND | ND | ND | ND | ND | ND | ND | ND | ND |
| 3 |  |  | ND | ND | ND | ND | ND | ND | ND | ND | ND |
| 4 | Female |  | ND | ND | ND | ND | ND | ND | ND | ND | ND |
| 5 |  |  | ND | ND | ND | ND | ND | ND | ND | ND | ND |
| 6 |  |  | ND | ND | ND | ND | ND | ND | ND | ND | ND |
| 1 | Male | 6.67 | ND | 43.1 | BLQ | ND | ND | ND | ND | ND | ND |
| 2 |  |  | ND | BLQ | ND | ND | ND | ND | ND | ND | ND |
| 3 |  |  | ND | BLQ | ND | ND | ND | ND | ND | ND | ND |
| 4 | Female |  | ND | ND | ND | ND | ND | ND | ND | ND | ND |
| 5 |  |  | ND | ND | ND | ND | ND | ND | ND | ND | ND |
| 6 |  |  | ND | ND | ND | ND | ND | ND | ND | ND | ND |
| 1 | Male | 33.3 | ND | 197 | 72.7 | ND | ND | ND | ND | ND | ND |
| 2 |  |  | ND | 91.9 | ND | ND | ND | ND | ND | ND | ND |
| 3 |  |  | ND | 61.6 | ND | ND | ND | ND | ND | ND | ND |
| 4 | Female |  | ND | 23.8 | ND | ND | ND | ND | ND | ND | ND |
| 5 |  |  | ND | 15.9 | ND | ND | ND | ND | ND | ND | ND |
| 6 |  |  | ND | 31.0 | 36.8 | 18.9 | ND | ND | ND | ND | ND |
| PTT = Prior to treatment  PTx = post treatment  ND = No peak detected  BLQ = Below limit of quantitation (10.0 ng/mL) | | | | | | | | | | | |

**Table S3. Plasma Concentrations of ceftobiprole (BAL9141)**

| **Animal ID** | **Sex** | **Nominal Ceftobiprole Dose (mg/kg)** | **Determined Concentrations (ng/mL)** | | | | | | | | |
| --- | --- | --- | --- | --- | --- | --- | --- | --- | --- | --- | --- |
|  |  |  | **PTT** | **5 min PTx** | **30 min PTx** | **1 hr PTx** | **2 hrs PTx** | **4 hrs PTx** | **6 hrs PTx** | **8 hrs PTx** | **12 hrs PTx** |
| 1 | Male | 1 | ND | 1530 | 1380 | 1060 | 621 | 282 | 120 | 60.2 | BLQ |
| 2 |  |  | ND | 753 | 1340 | 1030 | 461 | 247 | 63.9 | BLQ | BLQ |
| 3 |  |  | ND | 1750 | 654 | 956 | 591 | 235 | 102 | BLQ | BLQ |
| 4 | Female |  | ND | 1750 | 1180 | 1230 | 560 | 212 | 84.5 | BLQ | BLQ |
| 5 |  |  | ND | 1670 | 1480 | 954 | 473 | 152 | 68.1 | BLQ | BLQ |
| 6 |  |  | ND | 2080 | 1780 | 1030 | 631 | 247 | 74.3 | BLQ | BLQ |
| 1 | Male | 5 | ND | 9570 | 7350 | 4240 | 2400 | 1120 | 472 | 200 | 55.9 |
| 2 |  |  | ND | 8720 | 6000 | 4860 | 2470 | 999 | 439 | 177 | BLQ |
| 3 |  |  | ND | 8370 | 6310 | 4390 | 2460 | 986 | 508 | 186 | BLQ |
| 4 | Female |  | ND | 10100 | 6930 | 4870 | 2950 | 1120 | 464 | 174 | BLQ |
| 5 |  |  | ND | 8980 | 7090 | 4650 | 2350 | 915 | 309 | 137 | BLQ |
| 6 |  |  | ND | 8920 | 5830 | 3430 | 1610 | 590 | 202 | 90.0 | BLQ |
| 1 | Male | 25 | ND | 41100 | 35500 | 27200 | 11600 | 5630 | 2070 | 941 | 180 |
| 2 |  |  | ND | 35200 | 28800 | 24400 | 11500 | 4920 | 2040 | 875 | 225 |
| 3 |  |  | ND | 44600 | 32100 | 23700 | 15200 | 6150 | 3180 | 1070 | 301 |
| 4 | Female |  | ND | 48700 | 34600 | 29600 | 10100 | 6880 | 2340 | 754 | 183 |
| 5 |  |  | ND | 38900 | 28500 | 24600 | 9620 | 4760 | 1300 | 681 | 115 |
| 6 |  |  | ND | 49900 | 30800 | 22100 | 7870 | 3530 | 1010 | 437 | 79.9 |
| PTT = Prior to treatment  PTx = post treatment  ND = No peak detected  BLQ = Below limit of quantitation (10.0 ng/mL) | | | | | | | | | | | |

**Table S4.** **Simulated and measured PK parameters of ceftobiprole (BAL9141) in CMs and humans**

| **Species:** | | **CM mean** | | | | **Human mean (95% confidence interval)** |
| --- | --- | --- | --- | --- | --- | --- |
| **Ceftobiprole medocaril 2-hour IV infusion dose:** | | **1.33 mg/kg** | **6.67 mg/kg** | **20.0 mg/kg** | **33.3 mg/kg** | **666.67 mg** |
| **Cmax (µg/mL)** | **Simulated** | 2.3 | 11.5 | 34.4 | 55 | 22 (5.1-38.6) |
|  | **Measured** | 1.69 | 9.11 | - | 43.1 | - |
| **Free Cmax (µg/mL)** | **Simulated** | 1.3 | 6.3 | 19 | 30.4 | 18 (4.3-32.4) |
|  | **Measured** | 0.930 | 5.02 | - | 23.8 | - |
| **AUC (h*µg/mL)** | **Simulated** | 6.4 | 32 | 95.6 | 153 | 132 (11.2-285) |
|  | **Measured** | 4.8 | 24.2 | - | 121 | - |
| **Free AUC (h*µg/mL)** | **Simulated** | 3.5 | 17.6 | 52.7 | 84.4 | 111 (9.4-239) |
|  | **Measured** | 2.63 | 13.4 | - | 66.8 | - |

**Table S5. Simulated and measured free time over MIC of ceftobiprole (BAL9141) in CMs and humans**

| **Species:** | | **CM mean** | | | | **Human mean** |
| --- | --- | --- | --- | --- | --- | --- |
| **Ceftobiprole medocaril 2-hour IV infusion dose:** | | **1.33 mg/kg** | **6.67 mg/kg** | **20.0 mg/kg** | **33.3 mg/kg** | **666.67 mg** |
| **fT>MIC 4 µg/mL (h)** | **Simulated** | 0 (0%) | 2.5 (31%) | 4.2 (52.5%) | 5.2 (65%) | 9 |
|  | **Measured** | 0 (0%) | 0.73 (9.1%) | - | 4.9 (61.2%) | - |
| **fT>MIC 2 µg/mL (h)** | **Simulated** | 0 (0%) | 3.7 (46%) | 5.5 (68.7%) | 6.4 (80%) | 15 |
|  | **Measured** | 0 (0%) | 2.55 (31.9%) | - | 6.78 (84.8%) | - |
| **fT>MIC 1 µg/mL (h)** | **Simulated** | 2.2 (27.5%) | 4.9 (61%) | 6.7 (83.7%) | 7.6 (95%) | 22 |
|  | **Measured** | 0.106 (1.3%) | 4.34 (54.3%) | - | 8.22 (100%) | - |
| **fT>MIC 0.5 µg/mL (h)** | **Simulated** | 3.3 (41%) | 6.1 (76%) | 7.9 (98.7%) | 8.8 (100%) | >24 |
|  | **Measured** | 1.91 (23.9%) | 6.02 (75.2%) | - | 9.69 (100%) | - |

**Table S6. Individual animal challenge doses.**

| **Animal ID** | **Group** | **Sex** | **Total Inhaled Dose**  **(CFUs/CM)** |
| --- | --- | --- | --- |
| 7 | 1 | M | 522 |
| 8 | 1 | M | 838 |
| 9 | 1 | F | 417 |
| 10 | 1 | F | 482 |
| 11 | 1 | M | 452 |
| 12 | 1 | M | 728 |
| 13 | 1 | F | 1567 |
| 14 | 1 | F | 858 |
| 15 | 2 | M | 620 |
| 16 | 2 | M | 524 |
| 17 | 2 | M | 625 |
| 18 | 2 | F | 541 |
| 19 | 2 | M | 585 |
| 20 | 2 | M | 1162 |
| 21 | 2 | F | 355 |
| 22 | 2 | F | 480 |
| 23 | 3 | M | 566 |
| 24 | 3 | M | 815 |
| 25 | 3 | M | 563 |
| 26 | 3 | F | 685 |
| 27 | 3 | M | 605 |
| 28 | 3 | M | 1016 |
| 29 | 3 | F | 498 |
| 30 | 3 | F | 654 |
| 31 | 4 | M | 468 |
| 32 | 4 | M | 563 |
| 33 | 4 | F | 532 |
| 34 | 4 | F | 516 |
| 35 | 4 | M | 757 |
| 36 | 4 | M | 1044 |
| 37 | 4 | F | 938 |
| 38 | 4 | F | 1224 |


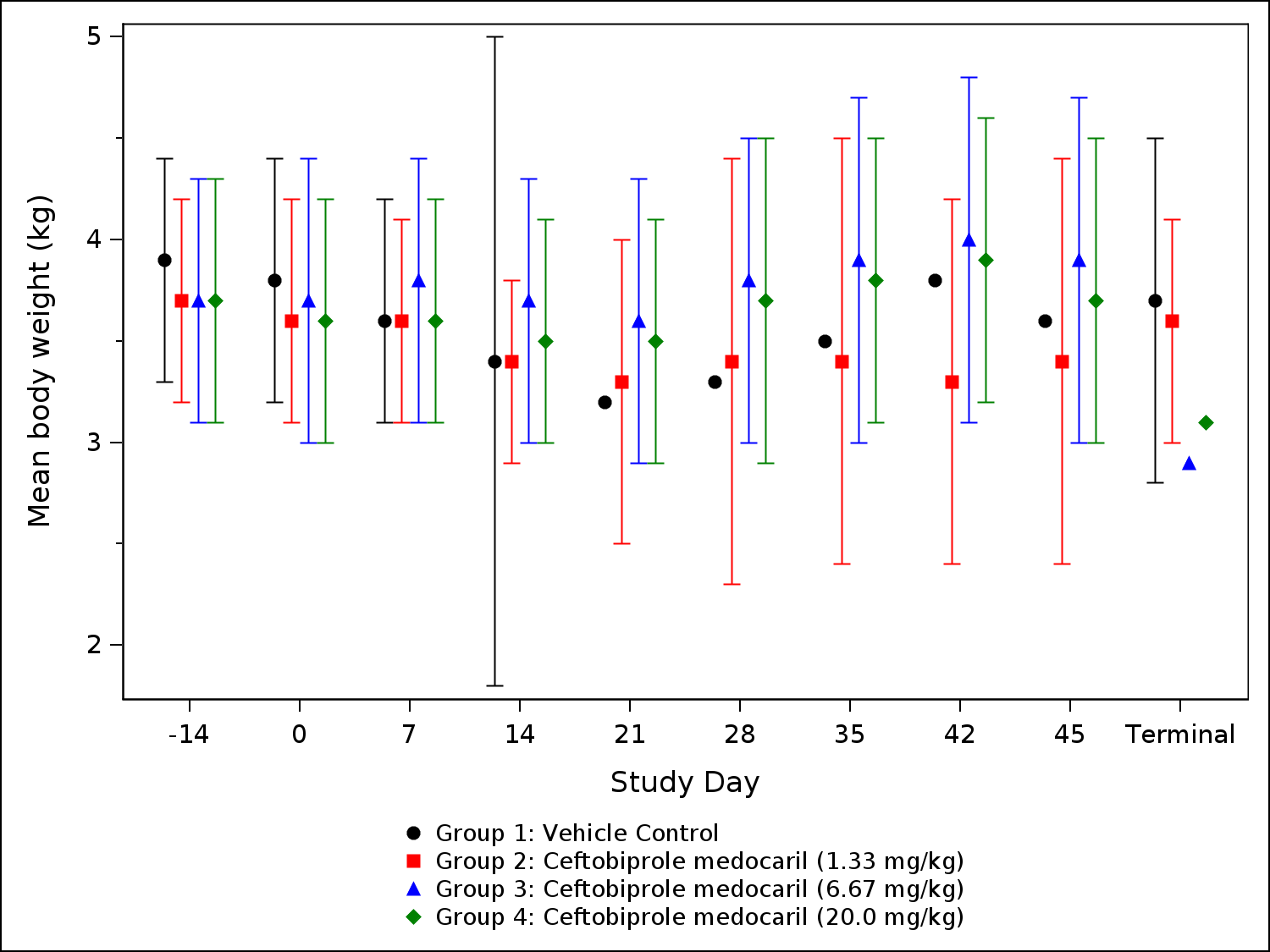


**Figure S1. Mean body weights and 95% confidence intervals.** Terminal represents body weights collected from CMs that terminated prior to 45 days post-challenge. Challenges were conducted on Day 0.


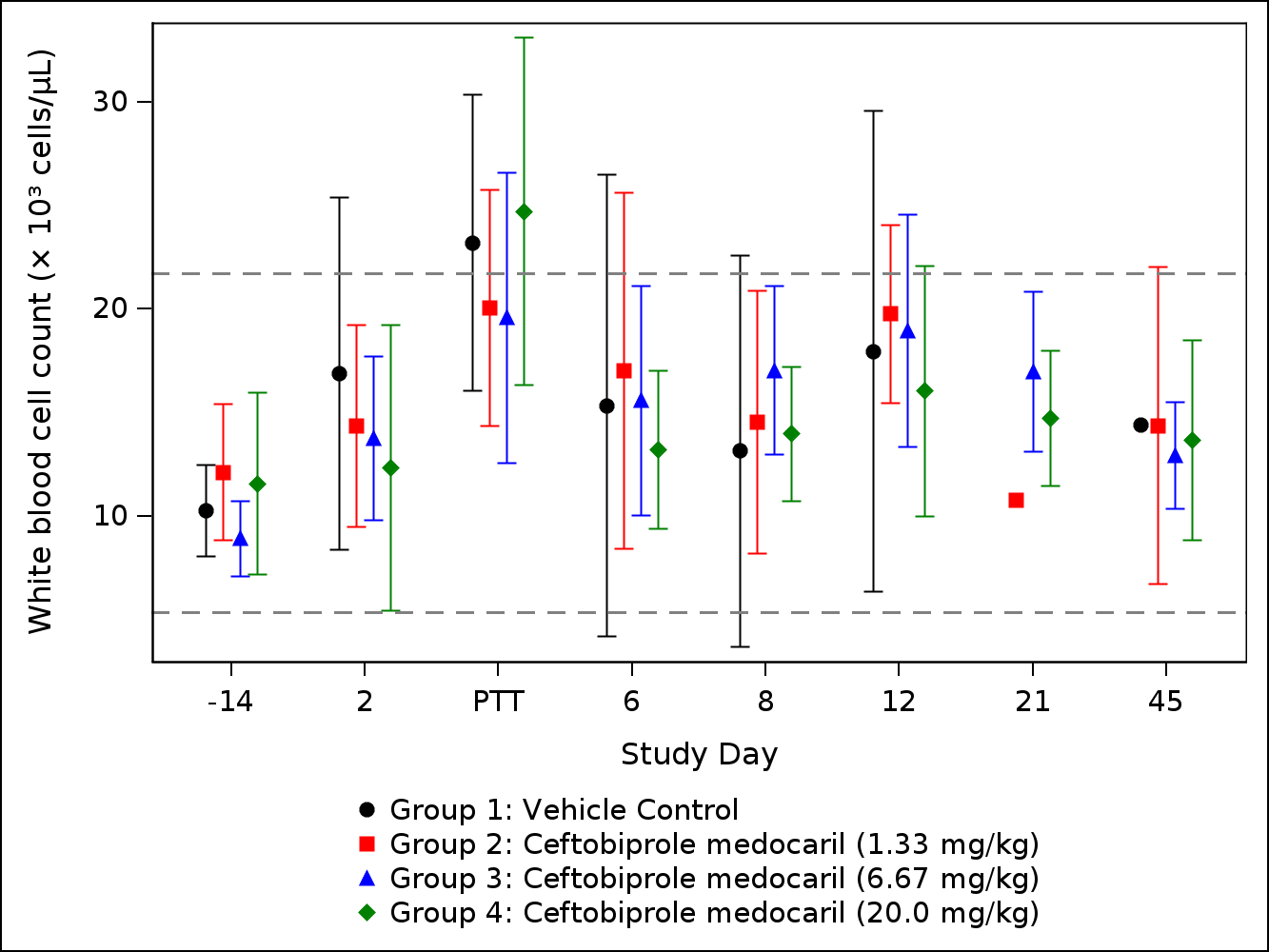


**Figure S2. Means and 95% confidence intervals for white blood cell counts.** Dashed gray lines represent the reference range of 5.34 – 21.72 × 10^3^ cells/µL. PTT: Prior to treatment.


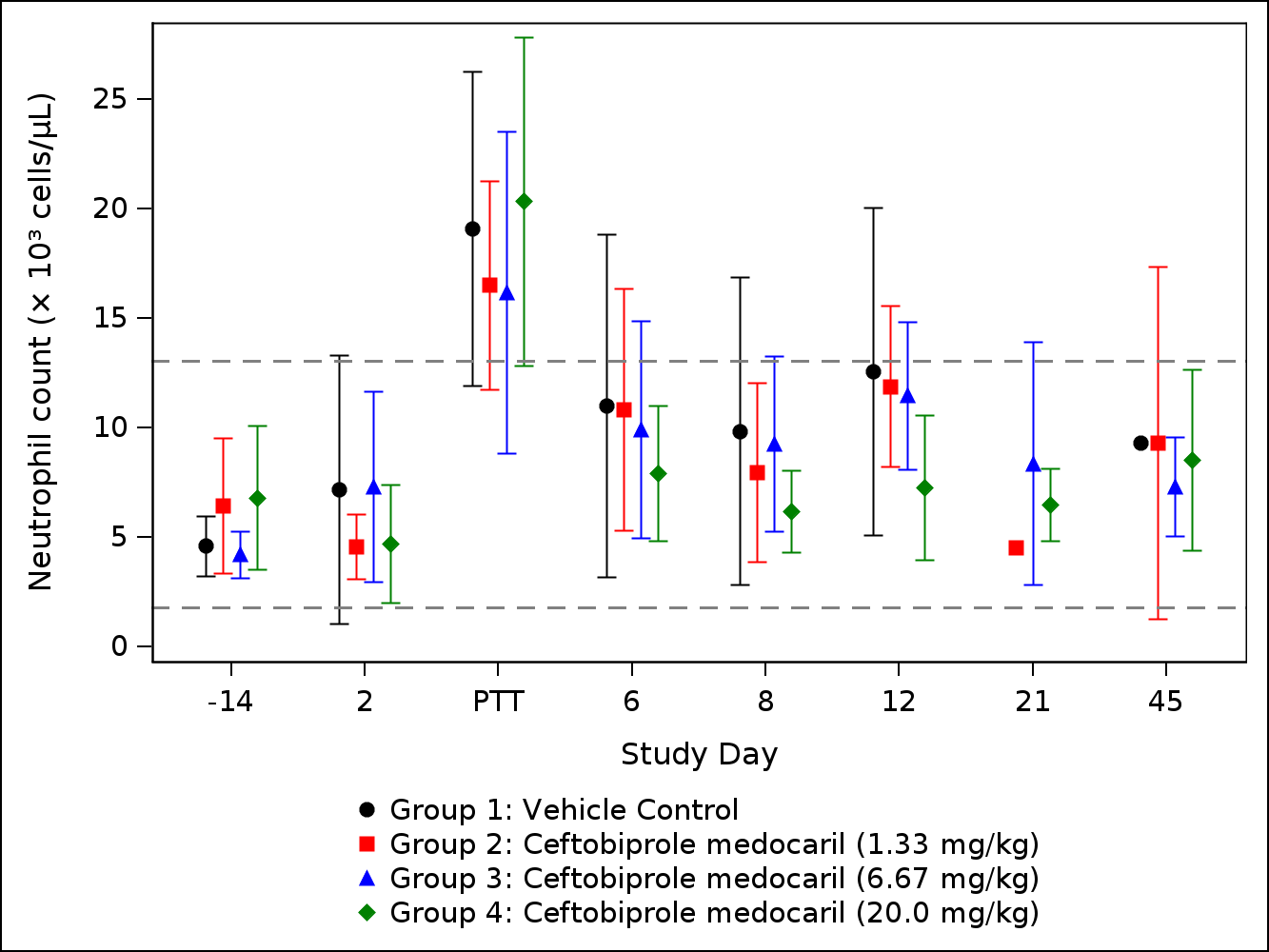


**Figure S3. Means and 95% confidence intervals for neutrophil counts.** Dashed gray lines represent the reference range of 1.78 – 13.05 × 10^3^ cells/µL. PTT: Prior to treatment.


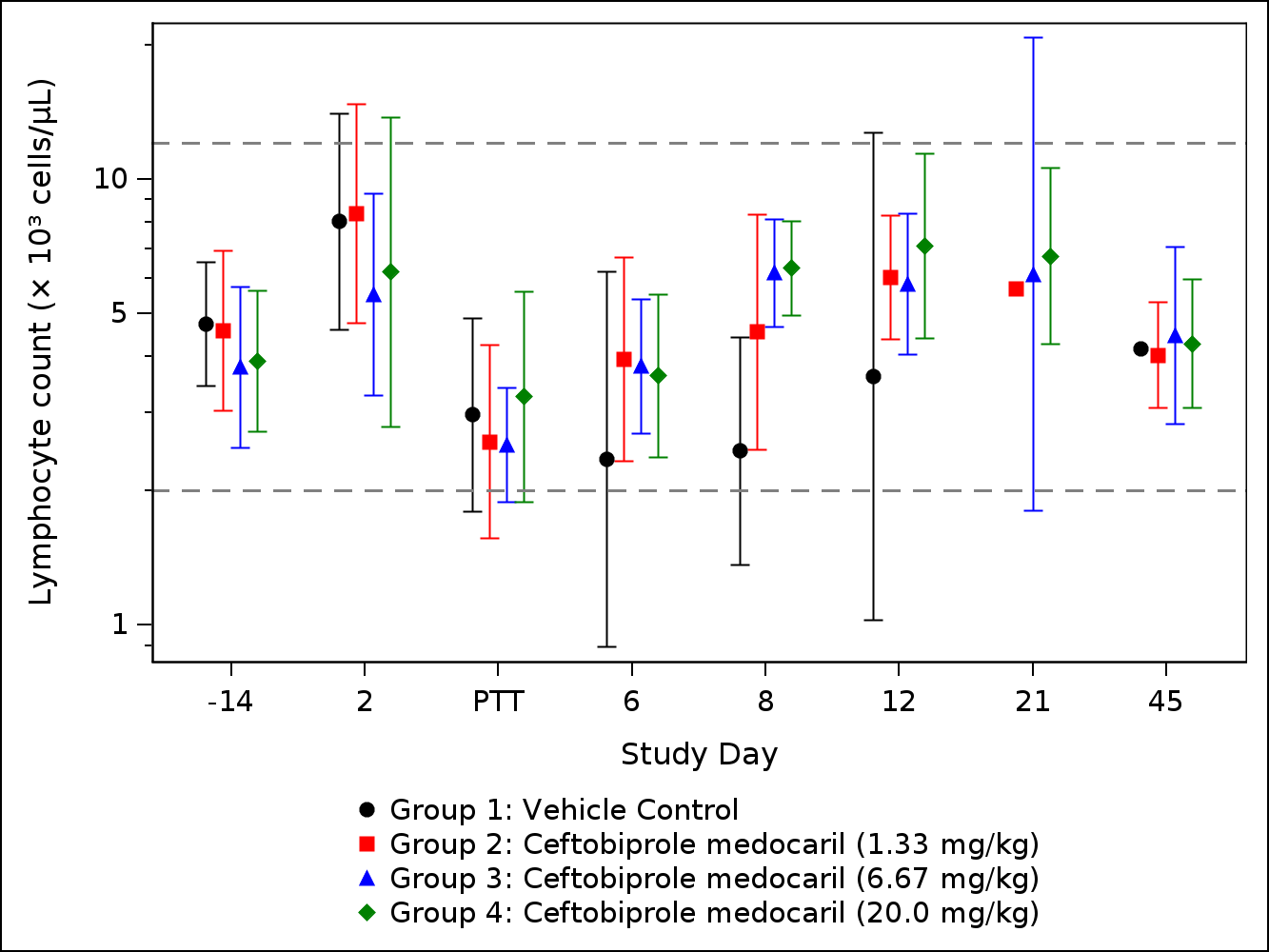


**Figure S4. Geometric means and 95% confidence intervals for lymphocyte counts.** Dashed gray lines represent the reference range of 2 – 12.06 × 10^3^ cells/µL. PTT: Prior to treatment. Lymphocyte counts were log-transformed for analysis and geometric means were calculated.


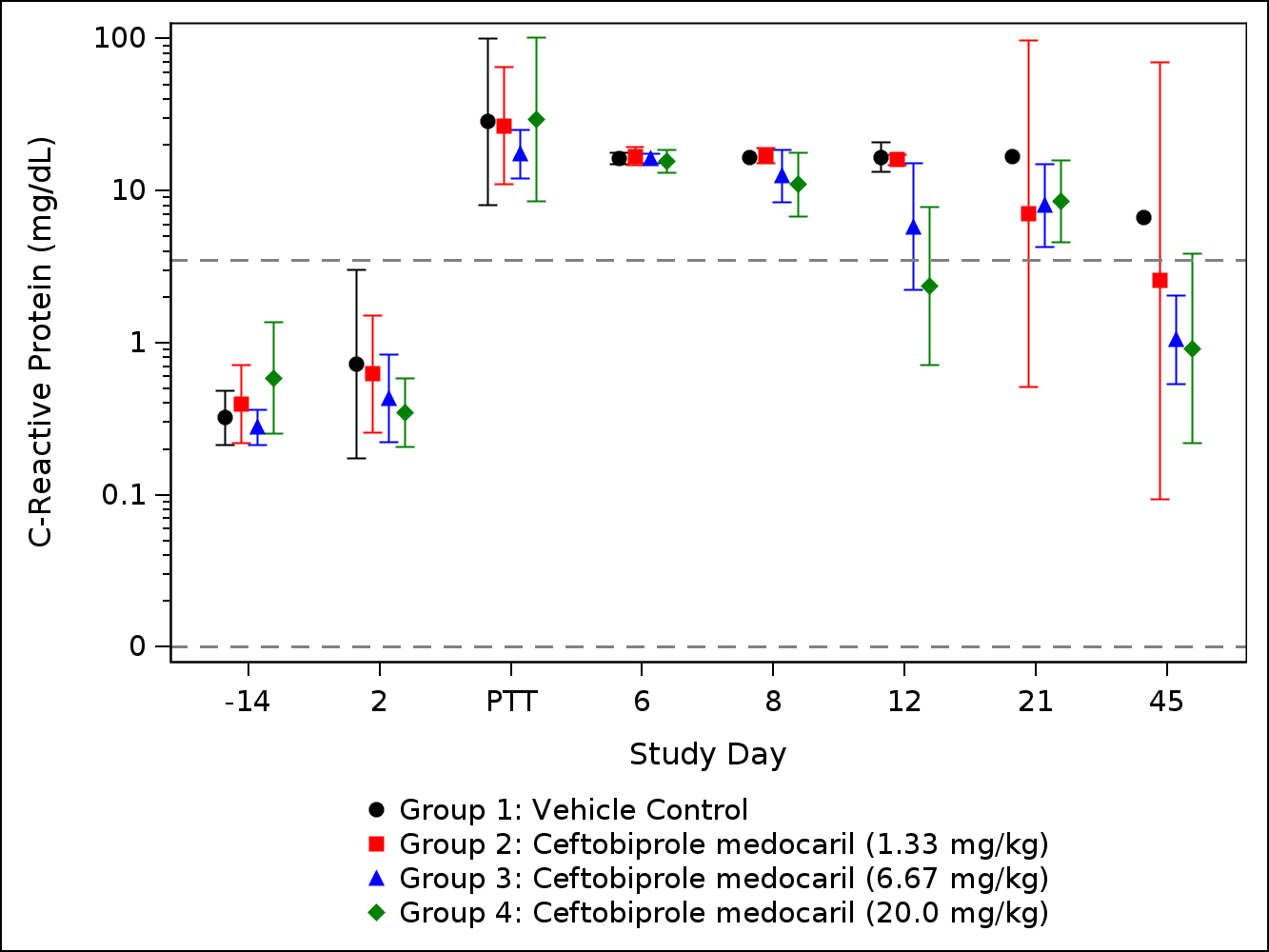


**Figure S5. Geometric means and 95% confidence intervals for CRP.** Dashed gray lines represent the reference range of 0 – 3.5 mg/dL. PTT: Prior to treatment. CRP was log-transformed for analysis and geometric means were calculated.


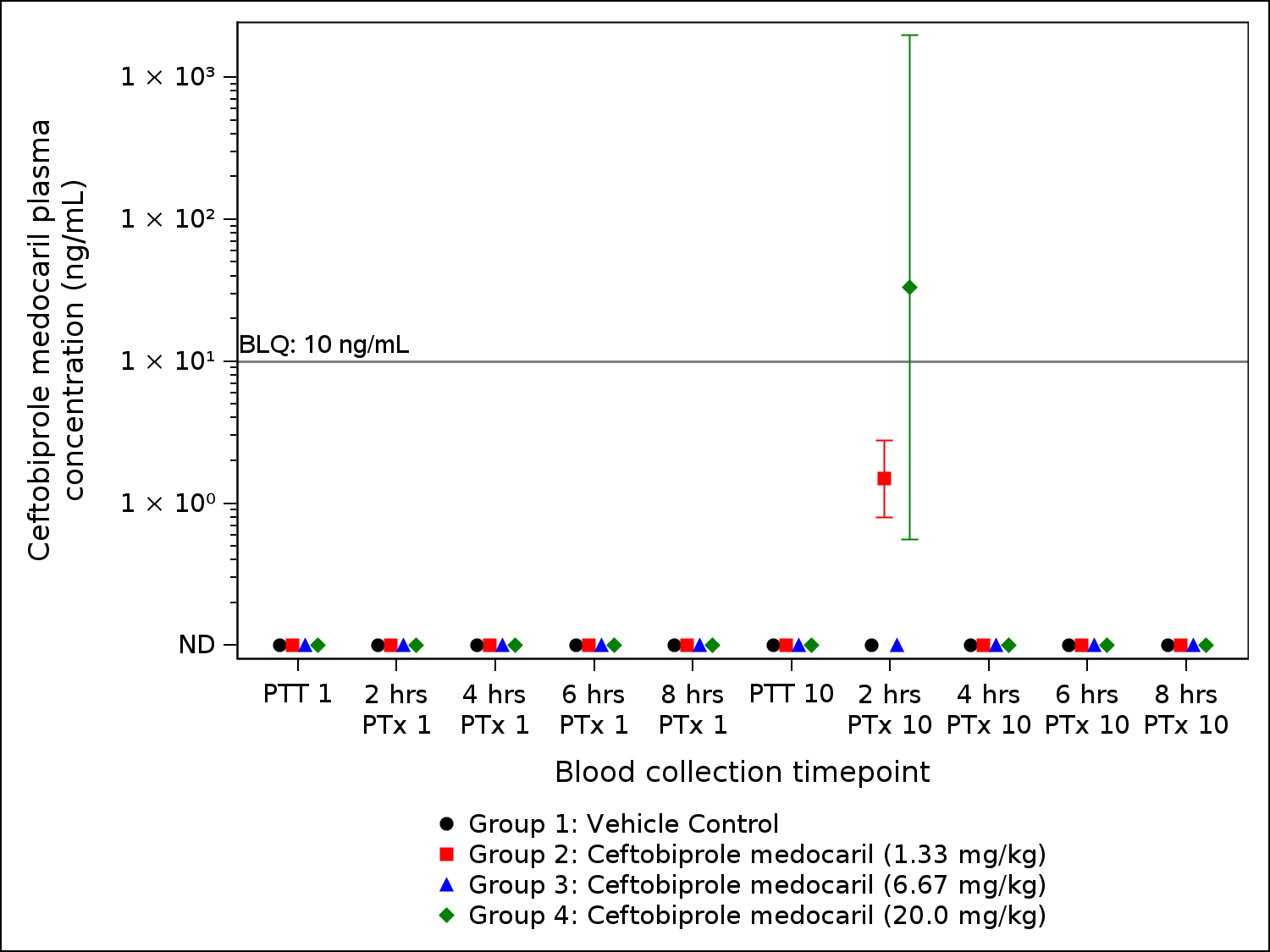


**Figure S6. Geometric mean ceftobiprole medocaril plasma concentration versus times in challenged CMs.** BLQ: Below the limit of quantification; ND: not detected; PTT 1: prior to treatment number 1; PTT 10: prior to treatment number 10; PTx 1: Post-treatment number 1; PTx 10: Post-treatment number 10. Group markers at each timepoint are slightly offset to distinguish between the groups (x-axis does not represent a time scale). Error bars represent 95% confidence interval.
